# A DNA methylation signature identified in the buccal mucosa reflecting active tuberculosis is changing during tuberculosis treatment

**DOI:** 10.1038/s41598-024-80570-4

**Published:** 2024-11-28

**Authors:** Isabelle Öhrnberg, Lovisa Karlsson, Shumaila Sayyab, Jakob Paues, David Martínez-Enguita, Mika Gustafsson, Patricia Espinoza-Lopez, Melissa Méndez-Aranda, Ericka Meza, Cesar Ugarte-Gil, Nicholas Kiprotich, Lameck Diero, Ronald Tonui, Maria Lerm

**Affiliations:** 1 Division of Inflammation and Infection, Lab 1, Floor 12, Linköping University, 58185 Linköping, Sweden; 2 Division of Infectious Diseases, Department of Biomedical and Clinical Sciences, Faculty of Medicine and Health Sciences, Linköping University, Linköping, Sweden; 3 Department of Physics, Chemistry and Biology, Linköping University, Linköping, Sweden; 4 Facultad de Medicina, Universidad Peruana Cayetano Heredia, Lima, Peru; 5 Instituto de Medicina Tropical Alexander Von Humboldt, Universidad Peruana Cayetano Heredia, Lima, Peru; 6 Laboratorios de Investigación y Desarrollo, Facultad de Ciencias e Ingeniería, Universidad Peruana Cayetano Heredia, Lima, Peru; 7 Department of Epidemiology, School of Public and Population Health, University of Texas Medical Branch, Galveston, TX USA; 8 Biochemistry and Clinical Chemistry, Moi University, Eldoret, Kenya; 9 AMPATH Kenya, Moi University, Eldoret, Kenya; 10 Department of Medicine, Moi University, Eldoret, Kenya; 11 Department of Pathology, Moi University, Eldoret, Kenya

**Keywords:** Tuberculosis, Treatment monitoring, Oral swabs, DNA methylation, Biosignatures, Buccal mucosa, Infectious-disease diagnostics, Tuberculosis, Bioinformatics, Diagnostic markers

## Abstract

Tuberculosis (TB) poses a significant global health threat, with high mortality rates if left untreated. Current sputum-based TB treatment monitoring methods face numerous challenges, particularly in relation to sample collection and analysis. This pilot study explores the potential of TB status assessment using DNA methylation (DNAm) signatures, which are gaining recognition as diagnostic and predictive tools for various diseases. We collected buccal swab samples from pulmonary TB patients at the commencement of TB treatment (n = 10), and at one, two, and six-month follow-up intervals. We also collected samples from healthy controls (n = 10) and individuals exposed to TB (n = 10). DNAm patterns were mapped using the Illumina Infinium Methylation EPIC 850 K platform. A DNAm profile distinct from controls was discovered in the oral mucosa of TB patients at the start of treatment, and this profile changed throughout the course of TB treatment. These findings were corroborated in a separate validation cohort of TB patients (n = 41), monitored at two and six months into their TB treatment. We developed a machine learning model to predict symptom scores using the identified DNAm TB profile. The model was trained and evaluated on the pilot, validation, and two additional independent cohorts, achieving an R^2^ of 0.80, Pearson correlation of 0.90, and mean absolute error of 0.13. While validation is needed in larger cohorts, the result opens the possibility of employing DNAm-based diagnostic and prognostic tools for TB in future clinical practice.

## Introduction

Tuberculosis (TB) is one of the infectious diseases that kills most people annually around the globe (1.3 million in 2022), with estimated increased death rates as a result of the COVID-19 pandemic due to undiagnosed and untreated TB cases^1^. TB is a preventable and curable disease, but if not treated the estimated TB fatality is high, between 20 and 70%^2^. The standard treatment for drug-susceptible TB (DSTB) includes isoniazid, rifampicin, pyrazinamide and ethambutol for six months^3^. Drug-resistant TB (DRTB) is an emerging problem worldwide affecting mainly low-income countries, with lower treatment success rates (63%) than for DSTB (88%)^1^. Smear microscopy and culture-based methods (culture conversion) of sputum samples are currently standard for TB treatment outcome measures and treatment monitoring of DSTB and DRTB^4^. No simple test to confirm cure after TB treatment exists. Sputum-based diagnostics are time- and resource-consuming and the procedure generates aerosols, which are biohazard risks and risks of contamination. For many patients, in particular children and HIV co-infected patients, producing sputum is difficult^5–7^. Another problem with sputum samples is that patients with HIV infection often have false negative results even with culture^8^. An alternative to conventional sputum sampling is sputum induction, which is a non-invasive method that requires trained personnel and may cause adverse events like nausea and headache^9^ and includes risks of transmission via aerosols^10^. Another way or acquiring a sample for diagnosis is via broncho-alveolar lavage using bronchoscopy, which is invasive, uncomfortable, costly and requires sedation of the patient^11^. Moreover, in many limited-resource settings there is no access to BSL3 laboratories^12^. Sputum smear microscopy has low sensitivity and specificity and cannot confirm the viability of the bacteria and genotypic and molecular tests for TB can generate false positive results after treatment. Taken together, there is a need for an easy test to identify TB and predict TB treatment outcome and the World Health Organization (WHO) is asking for new reliable and simple tools of TB treatment follow-up and monitoring^13^. A non-invasive, non-sputum-based test for *M. tuberculosis* would aid in the diagnosis and treatment monitoring of TB, particularly in limited-resource settings and for patients with HIV co-infection and for extrapulmonary disease. Recent studies have evaluated oral swab samples for the diagnosis of TB, with mixed indications of sensitivity and specificity for detecting *M. tuberculosis*^14–21^. DNA methylation (DNAm) is emerging as a clinically used diagnostic tool used in the field of oncology and has the potential to be used for several other medical conditions^22–25^. We have previously shown that mycobacteria give rise to DNAm changes in immune cells in blood and in the lung compartment^26–28^. Recently, we showed that TB disease and exposure can be identified based on DNAm signatures in buccal swabs^29^. Here, we collected buccal swab samples of patients with pulmonary DSTB from a pilot cohort in Peru at treatment start, and after one, two and six months of treatment. By analyzing the DNAm status of the buccal samples at treatment start in comparison with healthy controls, we identified a DNAm signature of TB that was altered during TB treatment. The signature was later applied on a validation cohort in Kenya for confirmation of the results. To our knowledge, this is the first study to investigate DNAm changes of TB patient buccal swab samples during TB treatment. DNAm analyses of buccal swab samples could have implications for TB diagnostics and treatment monitoring in the future.

## Results

### A distinct DNA methylation signature of active pulmonary TB in the buccal mucosa compared to healthy controls

**Fig. 1:**
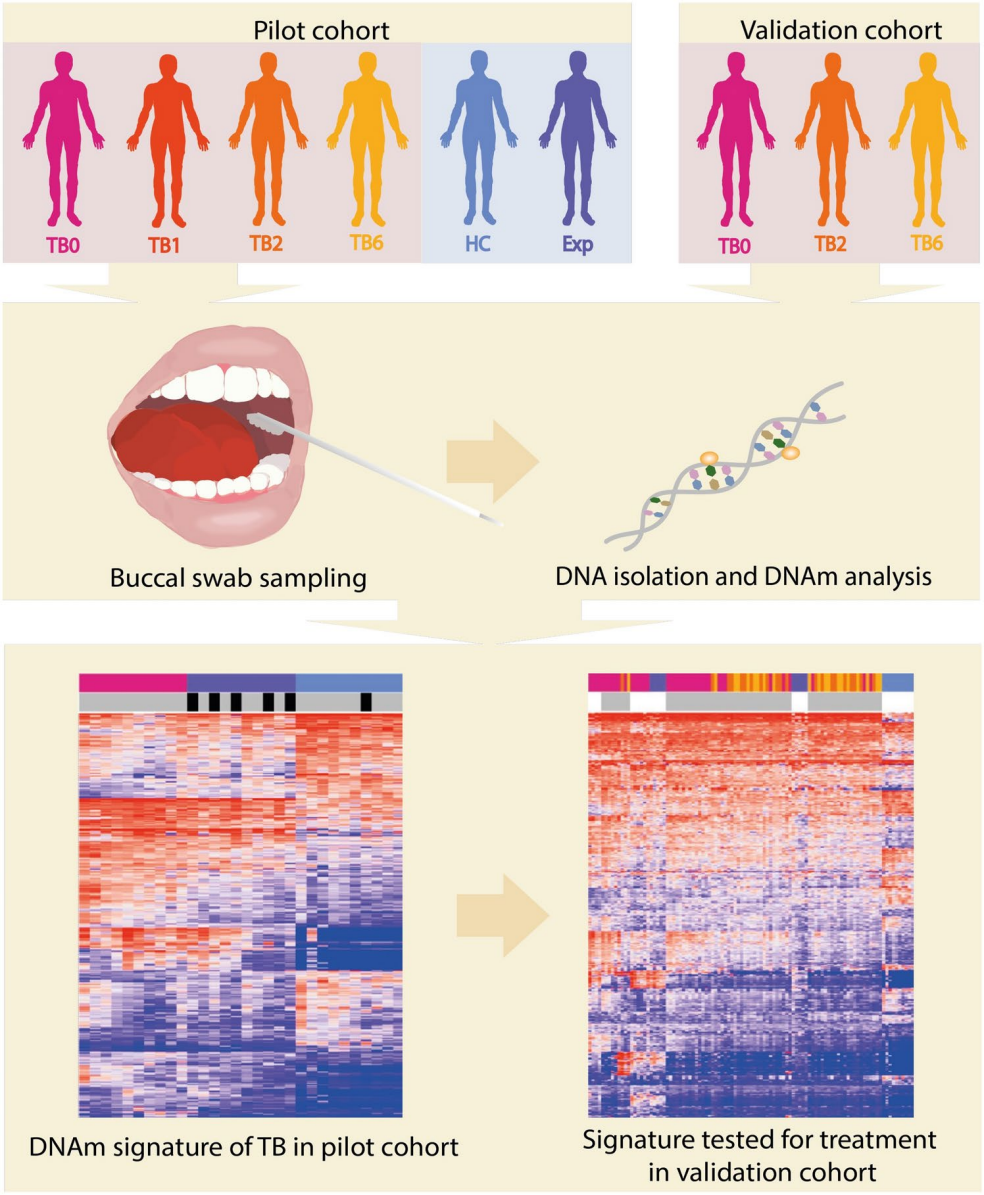
Overview of the study. Buccal swab samples were collected from TB patients at treatment start (TB0), at 1 month (TB1), 2 months (TB2), 6 months (TB6) of treatment and healthy controls (HC) and TB exposed (Exp) in a pilot cohort. DNA was isolated from the swab samples and DNA methylation (DNAm) analysis performed. Via computational analyses, a DNAm signature of TB was identified that was later tested for patients undergoing TB treatment in a validation cohort. TB, tuberculosis; TB0, TB patients at baseline; TB1, TB patients 1 month into treatment; TB2, TB patients 2 months into treatment; TB6, TB patients 6 months into treatment; HC, healthy controls; Exp, exposed; DNAm, DNA methylation.

**Fig. 2:**
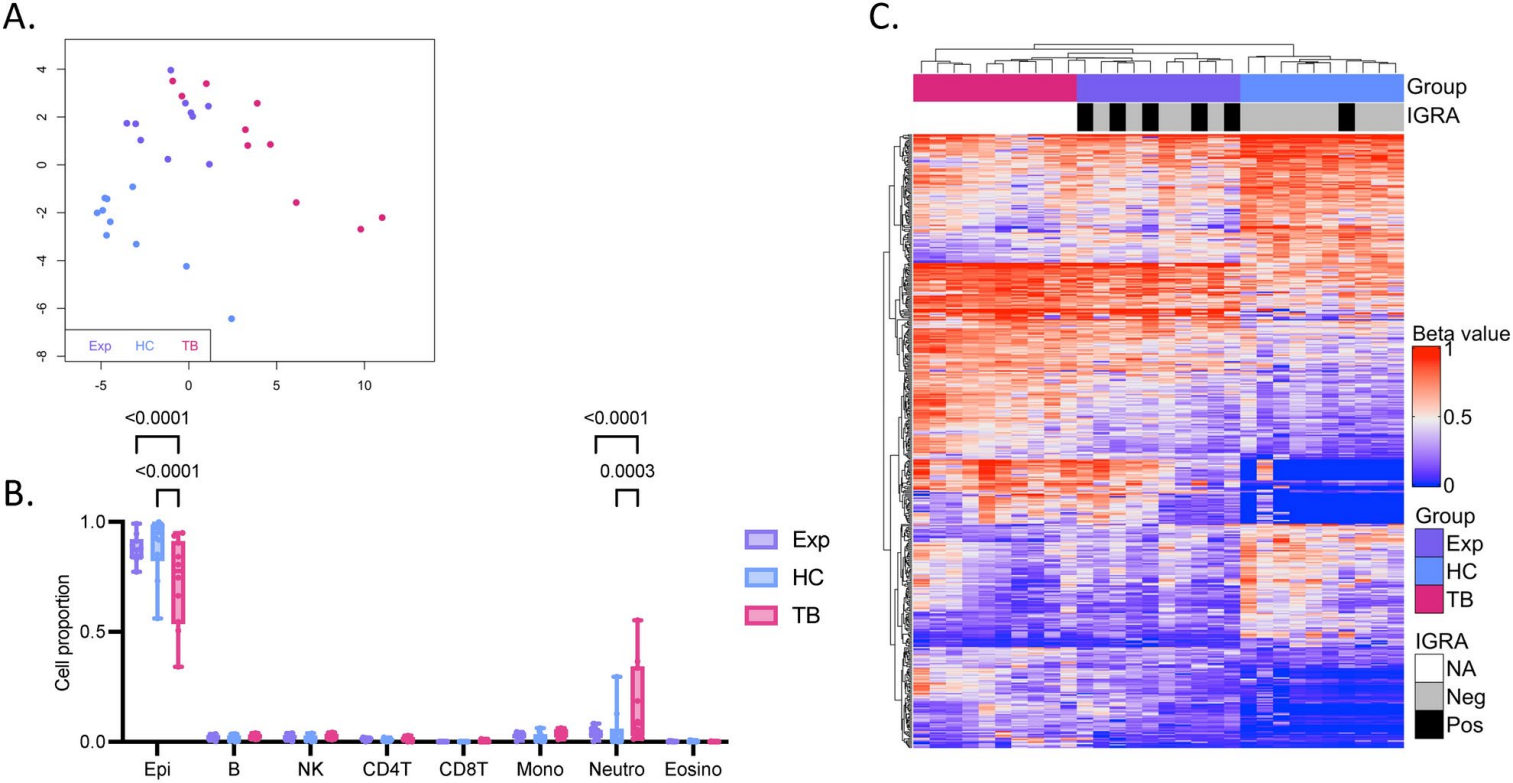
DNAm changes in the buccal mucosa of TB patients, TB exposed and healthy controls in pilot cohort. (**A**) A MDS plot of the 1,000 most variable CpG sites in the dataset (745,151 CpG sites) showing TB patients (TB) in pink, TB exposed (Exp) in purple and healthy controls (HC) in blue. (**B**) Cell proportions estimated using HepiDISH showing proportions of immune cells and epithelial cells in the groups. Significant difference in epithelial cells between TB and HC and Exp (*p* < 0.0001 and < 0.0001) and in neutrophils between TB and HC and Exp (*p* = 0.0003 and < 0.0001) (2-way ANOVA Tukey´s multiple comparisons test). (**C**) A heatmap of 468 DMCs (MMD ≥ 0.2, *p*.adj < 0.05) between TB and HC. Positive interferon-gamma release assay (IGRA) results for Exp and HC shown in black, and negative results shown in grey. DNAm, DNA methylation; TB, TB patients; Exp, tuberculosis exposed; HC, healthy controls; MDS, multidimensional scaling; DMCs, differentially methylated CpG sites; MMD, mean methylation difference; *p*.ajd, adjusted* p*-value; IGRA, interferon-gamma release assay.

See Fig. 1 for an overview of the study design. We included a pilot cohort of 10 pulmonary TB patients, 10 TB exposed contacts and 10 healthy controls and collected buccal swab samples for DNA isolation. The cohort was followed longitudinally with follow-up sampling at one, two and six months after inclusion. The demographics of the study participants are shown in Table 1. All TB patients were positive in sputum tests on GeneXpert MTB/RIF Ultra with drug sensitive TB and had a mean TB score of 4.5 ± 1.0 at treatment start corresponding to severity class 1 of 3^30,31^. To identify any inherent differences in the DNAm pattern of the buccal swabs of TB patients, TB exposed and healthy controls in the pilot cohort, we performed an unsupervised clustering using multidimensional scaling (MDS) on the dataset (745,151 CpG sites). We visualized the results in an MDS plot based on the 1,000 most variable CpG sites and observed separation between the active TB patients (TB), TB exposed (Exp) and healthy controls (HC) (Fig. 2A). To identify any differences in the cell proportions of the buccal swabs, we used the HepiDISH cell deconvolution algorithm. The analysis showed a significant difference in epithelial cell proportion between TB and Exp and HC (*p* < 0.0001 and *p* < 0.0001) and in neutrophils between TB and Exp and HC (*p* = 0.0003 and *p* < 0.0001) (Fig. 2B). Next, we investigated if there were any significant DNAm differences between TB and HC groups by identifying differentially methylated CpG sites (DMCs). We identified 468 DMCs (mean methylation difference, MMD ≥ 0.2; adjusted* p*-value, *(p*.adj) < 0.05), indicating that TB patients have a changed DNAm pattern in the oral mucosa. The beta values of DMCs were plotted in a heatmap including the Exp (Fig. 2C). The Exp and HC were tested with interferon-gamma release assay (IGRA) and five exposed subjects, and one healthy control tested positive. The IGRA status of HC and Exp are indicated in top annotations of the heatmap in black, the IGRA status did not influence the clustering of samples.

**Table 1:** Demographic characteristic data of Peruvian subjects.

Characteristics	TB (*n* = 10)	Exp (*n* = 10)	HC (*n* = 10)	*p*-value
Mean age year	44.6 ± 19.8	41.9 ± 12.9	28.9 ± 9.8	0.700
Mean BMI	21.8 ± 3.8	28.4 ± 4.1	25.9 ± 3.7	0.003*
*Sex*				0.126
Male	6 (60%)	2 (20%)	6 (60%)	
Female	4 (40%)	8 (80%)	4 (40%)	
*Disease phenotype*				
PTB	10 (100%)			
EPTB	0 (0%)			
*GeneXpert MTB/RIF Ultra*				
Positive in sputum	10 (100%)			
Positive in oral swab	2 (20%)			
*Smear microscopy*				
Positive	2 (20%)			
Paucibacillary	4 (40%)			
IGRA-positive		5 (50%)	1 (10%)	
*TB treatment*				
2RHZE/4RH	8 (80%)			
2RHZE/4RH + prolonged treatment	2 (20%)			
Smokers	0 (0%)	0 (0%)	0 (0%)	1.000
BCG vaccination (scar)	9 (90%)	9 (90%)	7 (70%)	0.142
HIV-positive	0 (0%)	0 (0%)	0 (0%)	1.000
Mean TB score 0 months	4.5 ± 1.0			

Continuous variables shown as mean ± standard deviation and significance testing using Mann–Whitney U-test, statistically significant results between Pat and Exp indicated with *. Categorical variables shown as n (%) and significance tested using Chi^2^. TB, tuberculosis patients; Exp, tuberculosis exposed; HC, healthy controls; BMI, body mass index; PTB, pulmonary tuberculosis; EPTB, extrapulmonary tuberculosis; IGRA, interferon-gamma release assay; 2RHZE/4RH, 2 months with rifampicin, isoniazid, pyrazinamide, ethambutol/4 months with rifampicin, isoniazid; BCG, Bacillus Calmette–Guérin.

### DNAm signature of active TB is enriched in infectious disease pathways

The DMCs identified as a TB signature was used in a pathway enrichment analysis. We used the 468 DMCs that were annotated to 242 genes in a disease module detection algorithm (DIAMOnD) and later an enrichment analysis using KEGG. We identified 264 significantly enriched pathways and the top 20 are shown in Supplementary Fig. 1, all pathways are listed in Supplementary List 1. The top enriched pathway was PI3K-Akt signaling pathway, which has been previously reported as a target for manipulation by intracellular *M. tuberculosis*^32^. We identified enrichment in several infectious disease pathways including Yersinia infection, Human Papillomavirus infection, Shigellosis, Salmonella infection and Human immunodeficiency virus 1 infection (Fig. S1). Bacterial invasion of epithelial cells was among the enriched pathways (gene count of 28, gene ratio of 0.1 and *p*.adj = 1.8e-20), see Supplementary List 1. Furthermore, several immune related pathways were amongst the top enriched, including Chemokine signaling pathway and T cell receptor signaling pathway. In summary, the distinct DNAm pattern identified in active TB patients was indicated to have implications in infectious diseases and immune responses. Additionally, we also explored the protein–protein interaction database STRING to find the biologically relevant pathways. Pathway analysis of the TB vs. HC dataset (468 DMCs) revealed "bacterial invasion of epithelial cells" as one of the top candidates KEGG pathways. This pathway aligns with known host–pathogen interactions in TB, where *M. tuberculosis* invades epithelial cells as part of its infection mechanism (STRINGdb:link: https://version-12-0.string-db.org/cgi/network?networkId=bbIMv5anjYVy). This finding was further confirmed using Funcoup PathwAX II analysis (FDR *p*-value 0.03 and 7.16e-13), suggesting that these DNAm changes might reflect the host’s response to bacterial invasion.

### Differentially methylated CpG sites between TB patients and healthy controls are altered during TB treatment

**Fig. 3:**
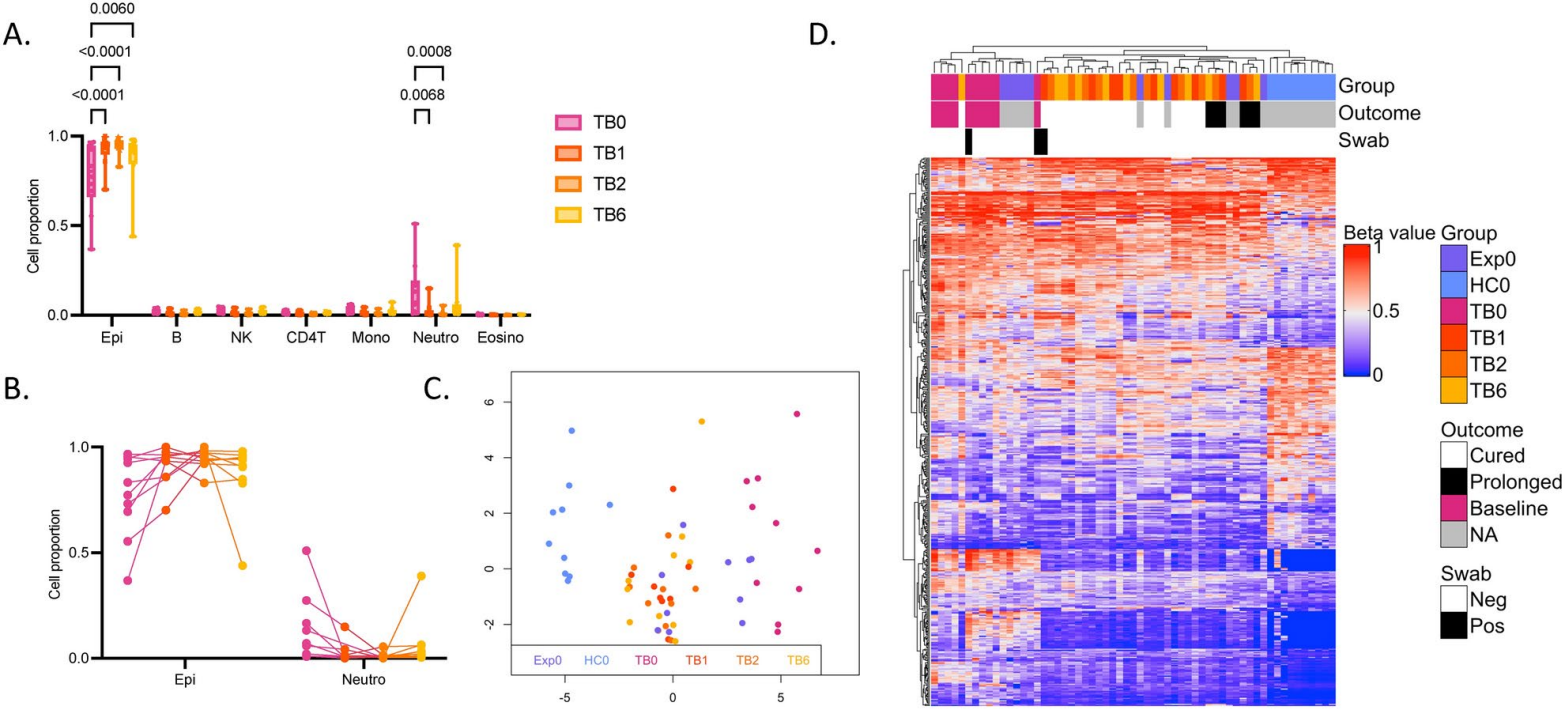
CpG sites differentially methylated between TB patients and healthy controls are altered during TB treatment. (**A**) Cell proportions estimated using HepiDISH showing proportions of immune cells and epithelial cells in the groups. Significant difference in epithelial cells between TB patients at baseline (TB0) and after one (TB1), two (TB2) and six (TB6) months of TB treatment (*p* < 0.0001, < 0.0001 and 0.006, respectively) and in neutrophils between TB0 and TB1 and TB2 (*p* = 0.0068 and 0.008, respectively) (2-way ANOVA Tukey´s multiple comparisons test). (**B**) Cell proportions of epithelial cells and neutrophils over time for each patient. (**C**) A MDS of 468 DMCs (MMD ≥ 0.2, *p*.adj < 0.05) identified between TB0 and healthy controls (HC0). The MDS is showing the TB patients followed during treatment TB1, TB2 and TB6 and TB exposed individuals (Exp0). (**D**) A heatmap of the 468 DMCs indicating treatment outcome (Outcome) and GeneXpert positivity in the buccal swabs (Swab) for TB patients in all timepoints. TB, TB tuberculosis; MDS, multidimensional scaling; DMCs, differentially methylated CpG sites; MMD, mean methylation difference; *p*.adj, adjusted* p*-value.

After identifying a changed DNAm pattern of TB patients compared to controls, we explored how the DNAm status of the buccal mucosa changed over time during TB treatment. First, we analyzed the cell proportions of the samples, showing significant increase in epithelial cells between TB patients at treatment start (TB0) and one, two and six months (TB1, TB2, TB6) into treatment (*p* < 0.0001, *p* < 0.0001 and *p* = 0.006, respectively) and a decrease in neutrophils between TB0 and TB1 and TB2 (*p* = 0.0068 and *p* = 0.008, respectively) (Fig. 3A). Figure 3B shows the longitudinal change in cell proportions of epithelial cells and neutrophils over time for each patient. One patient had increased neutrophils at baseline and one patient had increased neutrophils at the six months follow-up. Next, we wanted to explore how TB treatment affected the DNAm signature identified to reflect active TB. Using the 468 DMCs identified between the patients at baseline (TB0) and controls (HC), we performed a MDS including the longitudinal samples collected after one, two and six months of treatment (TB1, TB2, TB6) (Fig. 3C). The MDS show that the treated patients are separated from TB0, indicating that the DNAm alterations of the buccal mucosa seen in active TB are changing during TB treatment. Figure 3D represents a heatmap of the 468 DMCs, showing that treated patients and Exp cluster between the HC and TB0, representing a spectrum from active TB to exposed and treated patients to healthy controls. All TB patients received standard treatment for drug-sensitive TB (two months with isoniazid, rifampicin, pyrazinamide, ethambutol; four months with isoniazid and rifampicin (2RHZE/4RH) for a total of six months). Two patients received prolonged treatment due to abnormalities in the follow up chest X-rays. The treatment outcome is indicated in the heatmap and the two participants receiving prolonged treatment are clustering closer to the HC0 compared to TB0. The collected buccal swabs were also tested with GeneXpert MTB/RIF Ultra for detection of bacteria in the oral cavity and two patients had positive results at baseline and one persisted positive at the one month follow-up. These patients are marked in the top bar of the heatmap, the patient positive at the one month follow-up cluster close to the active TB group. One TB6 patient clustering together with the TB0 group in the heatmap (Fig. 3D), was also identified as an outlier of cell proportions in the buccal mucosa (Fig. 3B), with a higher neutrophil proportion at six months. The HC and Exp were also followed over six months and the longitudinal DNAm changes in these groups at baseline and one, two and six months are shown in Supplementary Fig. 2A. Furthermore, 468 DMCs identified between TB0 and HC0 did not show longitudinal changes in the HC and Exp groups (Fig. S2B).

### The TB signature of the pilot cohort changed during TB treatment in independent validation cohort

**Fig. 4:**
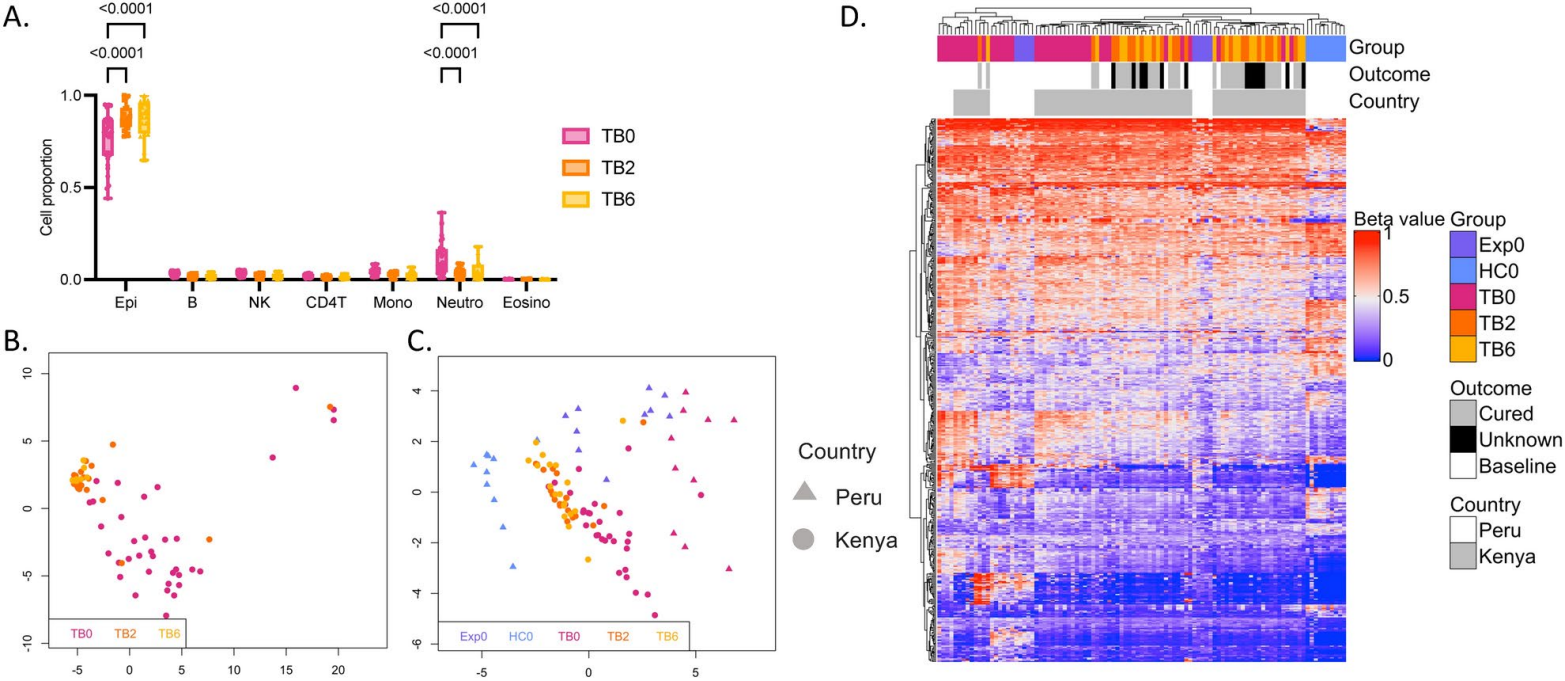
Validation of longitudinal DNAm changes in the buccal mucosa during TB treatment in an independent cohort. (**A**) Cell proportions estimated using HepiDISH showing proportions of immune cells and epithelial cells in the different timepoints. Significant difference in epithelial cells between TB patients at baseline (TB0) and after 2 (TB2) and 6 (TB6) months of treatment (*p* < 0.0001 and < 0.0001, respectively) and in neutrophils between baseline and two and six months of treatment (*p* < 0.0001 and < 0.0001, respectively) (2-way ANOVA Tukey´s multiple comparisons test). (**B**) A MDS plot of the 1,000 most variable CpG sites in the dataset of the validation cohort (744,330 CpG sites) showing TB0 in pink, TB2 in orange and TB6 in yellow. (**C**) MDS plot of 468 DMCs (mean methylation difference (MMD) ≥ 0.2, *p*.adj < 0.05) identified between active TB patients at baseline (TB0) and healthy controls (HC0) in the pilot cohort. The MDS is showing all baseline samples from the pilot cohort (Peru, triangle) and the TB patients from the validation cohort (Kenya, circle) followed during treatment after two and six months (TB2 and TB6). (**D**) A heatmap of the 468 DMCs indicating group, treatment outcome and country by colour in top bar (Peru in white and Kenya in grey).

To validate our findings, a Kenyan cohort of 41 patients with TB were included in the study and followed up after two and six months of treatment. Demographics of the validation cohort are shown in Table 2. The patients had a mean TB score of 5.9 ± 2.2 at treatment start, corresponding to severity class 2^31^, which was higher compared to the pilot cohort. We performed the same analyses of the DNAm data from the buccal swab samples. We first explored the longitudinal cell proportion changes in the different timepoints of TB treatment. All patients in the validation cohort received standard treatment for drug-sensitive TB (2RHZE/4RH). In line with the findings in the pilot cohort, we identified significant increase of epithelial cells between active TB patients (TB0) compared to after two (TB2) and six (TB6) months of treatment (*p* < 0.0001 and *p* < 0.0001, respectively) and decrease in neutrophils between TB0 and TB2 and TB6 (*p* < 0.0001 and* p* < 0.0001, respectively) (Fig. 4A). Next, we created a MDS plot based on the top 1,000 most variable CpG sites in the dataset of the pulmonary TB patients of the validation cohort (744,330 CpG sites), showing TB6 clustered as one distinct group separated from TB0 (Fig. 4B). Most of the patients two months into treatment clustered with the TB6 group. Seven (17%) patients were coinfected with HIV and this did not influence the clustering of samples in the MDS plot (Fig. S3A). After identifying a signature reflecting active TB in the pilot cohort, we investigated how these DMCs changed during TB treatment in the validation cohort. Figure 4C shows a MDS of the 468 identified DMCs including all baseline samples from the pilot cohort (triangle) and the longitudinally collected TB patient samples from the validation cohort (circle). Longitudinal changes during TB treatment were confirmed in the validation cohort as TB2 and TB6 samples cluster separately from the active TB patients (TB0). The 468 DMCs were also plotted in a heatmap showing that the active TB patients of the validation cohort are grouping together with the active TB patients of the pilot cohort and that the Kenyan treated patients (TB2 and TB6) cluster together with the Exp0 between the TB0 and HC0 groups (Fig. 4D). The treatment outcome is indicated in the top bar of the heatmap with 15 patients documented as cured and 12 patients with unknown outcome or lost to the six-month follow-up represented in the plot. Generally, the BMI of the patients increased during treatment and their symptoms decreased (Supplementary Table 1). In the validation cohort, five patients with extrapulmonary TB were included (Supplementary Table 2). Adding these patients to the heatmap showed that the disease phenotype did not influence the clustering of the patients (Fig S3B). In summary, the analysis confirms that active TB patients have a distinct DNAm pattern compared to healthy controls and exposed and that TB treatment alters this DNAm signature.

**Table 2:** Demographic characteristic data of Kenyan validation cohort.

Characteristics	TB (*n* = 41)
Mean age year	33.9 ± 13.2
Mean BMI	18.5 ± 4.1
*Sex*	
Male	27 (66%)
Female	14 (34%)
*Disease phenotype*	
PTB	35 (85%)
EPTB/ND	6 (15%)
*Diagnostic method*	
Sputum GeneXpert	27 (66%)
Smear microscopy	6 (15%)
U-LAM	7 (17%)
MRI	1 (2%)
Smokers	10 (24%)
BCG vaccination	38 (93%)
HIV-positive	7 (17%)
Mean TB score 0 months	5.9 ± 2.2

Continuous variables shown as mean ± standard deviation. Categorical variables shown as n (%); TB, tuberculosis patients; BMI, body mass index; PTB, pulmonary tuberculosis; EPTB, extrapulmonary tuberculosis; ND, no data; U-LAM, urine-lipoarabinomannan; MRI, Magnetic resonance imaging; BCG, Bacillus Calmette–Guérin.

### DNAm changes in the buccal mucosa during TB treatment

We continued to investigate longitudinal DNAm changes during TB treatment in the patients by comparing the patients at baseline and after 6 months of treatment. In the pilot cohort, we observed significant differences (epithelial and neutrophil) in the cell type proportions (Fig. 3A). This was taken into account by using them as covariates in the linear model. When difference in cell proportions was used as covariates in the DMC identification model for the pilot cohort, it resulted in 0 DMCs. In an alternative approach, we did not use the cell type as covariates in the model for the pilot study and identified 56 DMCs (MMD ≥ 0.2, *p*.adj < 0.05), shown in a heatmap in Supplementary Fig. 4A, demonstrating that the DNAm status in the buccal mucosa was different for patients at treatment start, and after one, two and six months of TB treatment. This could be due to the correlation between the early response to TB and cell type proportions that may be confounded and cannot be separated from the study groups in this case. The two patients receiving prolonged treatment are indicated in the top bar. Two patients had buccal swabs positive in GeneXpert analysis (one patient was positive at TB0 and one at TB0 and TB1). These patients cluster among the other patients in the heatmap (Fig. S4A). Next, we investigated if there were any significant DNAm changes between TB0 and TB6 in the notably larger validation cohort. Here, we identified a TB treatment signature of 99 significant DMCs, using a more robust analysis accounting for the differences in cell types. The DMCs are plotted in a heatmap in Supplementary Fig. 4B. The treatment outcome and HIV coinfection status is indicated in the top dendrogram of the heatmap. HIV coinfected patients clustered among the rest of the patients in the heatmap of the 99 DMCs (Fig. S4B). We investigated the similarities of the TB treatment signatures (TB0 vs TB6) identified in the pilot cohort and the validation cohort in a Venn analysis and did not find any overlap (Fig. S5). Furthermore, we compared the treatment signatures with the TB disease signature identified in the pilot cohort (TB0 vs HC) and found 3 overlapping DMCs (Fig. S5).

In order to explore the biological mechanisms of the DNAm changes found during the TB treatment, we performed the pathway analysis using the database STRING. The pathway analyses of DMCs in pilot cohort comparing baseline TB to 6 months treatment, identified the “Glutamatergic synapse” pathway as the top candidate with an FDR *p*-value of 9.93e-06. Literature supports a connection between glutamatergic signaling and host defense mechanisms against Mtb infection^33^, pointing to a possible role for these epigenetic changes in the host’s immune response to TB. Similarly, in the validation cohort (99 DMCs), the "Hippo signaling pathway" (FDR *p*-value 0.002) and "sensory system disease" pathways were highlighted as top candidates^34^.

### DNAm changes in peripheral blood mononuclear cells during TB treatment

In the Kenyan validation cohort, we also collected peripheral blood mononuclear cells (PBMCs) at each timepoint and investigated the DNAm changes over time. First, we performed a cell deconvolution analysis showing differences in the proportions of B cells, NK cells, CD4 T cells, monocytes and neutrophils between TB0, TB2 and TB6 (Fig. S6A). We observed a relative increase in B cells and CD4 T cells and decrease in monocytes and neutrophils over time. Next, we investigated if there were any inherent DNAm differences in the PBMCs between the groups using unsupervised clustering with MDS. In line with the findings from the buccal mucosa, we identified clustering of TB0 samples, and no separation of samples collected after two or six months of treatment and that HIV coinfection did not separate patients (Fig. S6B). We further added the five patients with extrapulmonary TB to the analysis and did not see any clustering based on the disease phenotype in the PBMCs (Fig. S6C). To investigate if there were any statistically significant differences between the groups, we investigated the longitudinal changes between the timepoints TB0 and TB6 for differential methylation but no significant DMCs (MMD ≥ 0.2, *p*.adj < 0.05) could be identified.

### A machine learning model trained on selected CpG sites achieves accurate symptom score predictions

**Fig. 5:**
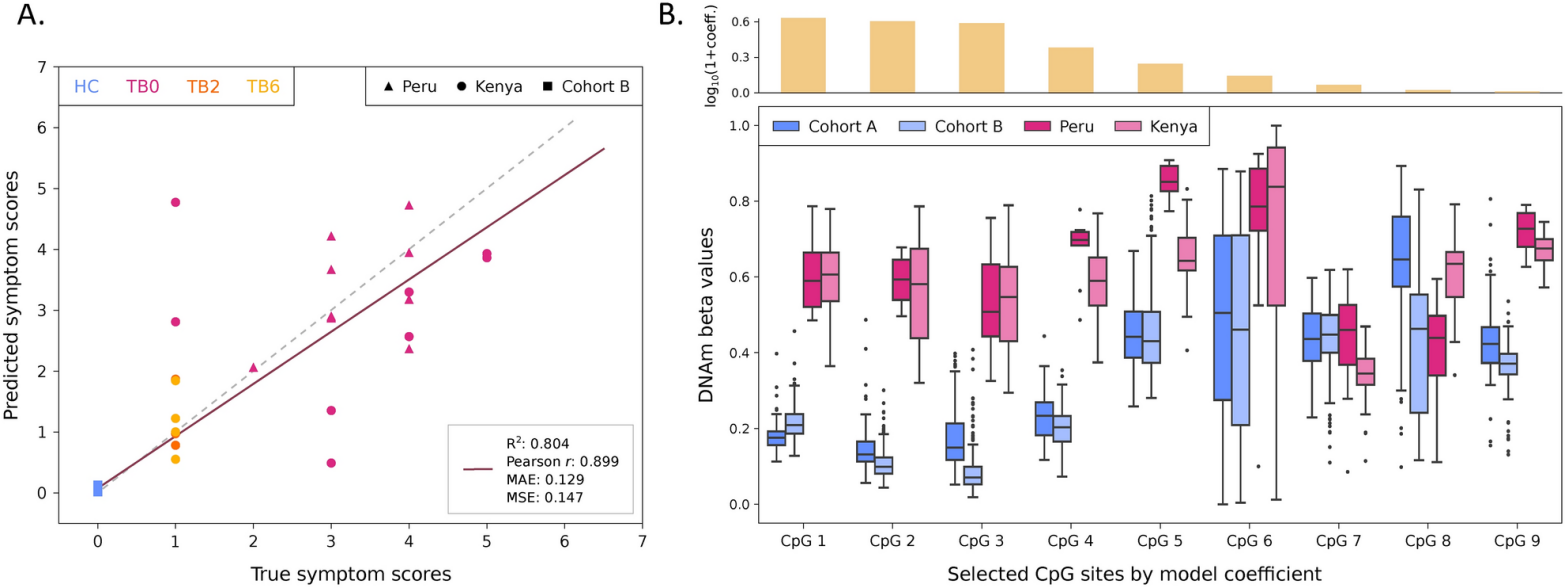
Performance evaluation of the symptom score predictive model and methylation beta values of the selected CpG sites. (**A**) Scatter plot of the predicted vs. true symptom scores of test set samples from the Kenyan cohort, baseline TB patients from the Peruvian cohort, and healthy individuals from the external validation cohort B (*n* = 276) obtained from the elastic net model trained with optimal hyperparameters. The regression line (purple, solid) shows the best fit to the data, while the identity line (grey, dashed) represents the perfect match between predicted and true values. Performance is evaluated using R-squared (R^2^), Pearson correlation, mean absolute error (MAE), and mean squared error (MSE). (**B**) Box plots of the DNA methylation beta values of the nine CpG sites with non-zero coefficients on the final model for the healthy external validation cohorts A and B, and the baseline TB patients from the Kenyan and Peruvian cohorts (*n* = 96, 250, 36, 10, respectively). The top bar plot indicates the log10 of (1 + coefficient) of each selected CpG site, representing their contribution to the model’s predictions.

To assess the robustness of the identified TB-associated DMCs across populations and their disease specificity, we implemented a machine learning approach to train and evaluate a regression model for the prediction of symptom scores (here defined as self-reported symptoms including cough, hemoptysis, dyspnea, night sweat, chest pain, BMI < 16, BMI < 18). For this purpose, we used both the Kenyan and Peruvian cohorts, as well as two external validation datasets of buccal swab DNAm profiles (referred to as A and B). First, we selected the most relevant CpG sites from the 468 DMCs identified between TB0 patients and HC from the Peruvian cohort by training an elastic net model using fivefold cross-validation (CV) on Kenyan cohort samples. To avoid data leakage, individual-level grouping was employed to ensure that samples from the same patient at different time points were not used in both training and test sets. This process yielded 99 unique CpG sites with a non-zero coefficient in at least one fold, of which 13 CpGs were consistently present across all folds, demonstrating their predictive utility for symptom score estimation. For model selection, eight linear and non-linear regressors were evaluated, conducting hyperparameter optimization (Methods). We chose an elastic net regression model with alpha of 1.63e-3 and L1 ratio of 0.1. Thus, we re-trained this model with best hyperparameters on the selected 13 CpGs from the Kenya training set (including TB patients at 0, 2, and 6 months post-treatment, *n* = 66) combined with healthy controls from the external cohort A (*n* = 96), and evaluated it on the Kenya test set (*n* = 16), Peru cohort TB0 patients (*n* = 10), and healthy controls from the external cohort B (*n* = 250). The final symptom score regressor achieved highly accurate predictions (Fig. 5A), with an overall R^2^ of 0.80, Pearson* r* of 0.90 (*p* = 1.63e-100), and mean absolute error (MAE) of 0.13 across the combined test sets, highlighting the generalizability of the model to different populations. The mean ± standard deviation (SD) symptom score estimated for external cohort B healthy controls was 0.05 ± 0.02 (assigned symptom score = 0), while predictions for TB patients were significantly correlated with their true scores (Pearson *r* = 0.57, p = 2.34e-3, MAE = 0.89), with estimated symptom scores of 2.07 ± 1.29 (true average score = 2.13 ± 1.54) for the Kenyan, and 3.28 ± 0.80 (true average score = 3.30 ± 0.64) for the Peruvian cohort. Further analysis of the model coefficients revealed that nine CpG sites, associated with seven genes, had non-zero coefficients in the final model (Fig. 5B). The DNAm beta values for these CpGs displayed consistent patterns among TB0 patients from both Kenya and Peru, which were distinct from those observed in healthy controls from external cohorts A and B. This distinction underscores the relevance of the identified sites for TB at disease onset. In sum, our machine learning model trained on selected CpG sites effectively predicts TB symptom scores across multiple populations, providing a robust framework for future studies aiming to translate epigenetic markers into clinically actionable biomarkers for TB diagnosis and prognosis.

## Discussion

An expansion of the current toolkit for diagnosis, prevention and treatment is essential for reaching the United Nations’ Sustainable Development Goals for 2030 of ending the TB epidemic^1,35^. DNAm analysis of buccal cells derived from oral swab samples have previously been used for biomarkers of rheumatoid arthritis and obesity^36,37^. We have identified DNAm alterations in blood and lung-derived immune cells in individuals exposed to TB^27,38^. In recent work we have also observed that epigenetic changes of TB occur in oral mucosa^29^. Previous studies have not included temporal data and only captured the DNAm status of one time point. Here, we show a distinct DNAm signature in the buccal mucosa of active pulmonary TB patients at treatment start, which is changed during TB treatment. We demonstrate that the DNAm pattern in the oral mucosa is different for patients that just started TB treatment in comparison to patients that have undergone treatment for one to six months. The results were validated in an individual cohort of patients with pulmonary and extrapulmonary TB. The TB phenotype and the HIV coinfection status did not affect the results in the validation cohort, which implies that oral swabs could be used even for extrapulmonary TB and HIV coinfected patients. Even though treatment outcome was not registered for all patients in the validation cohort, the decrease in symptoms in the two- and six-month follow-ups indicates that treatment was successful, suggesting that following DNAm changes in the oral mucosa during treatment can be used for treatment monitoring. The TB score severity class was higher in the Kenyan cohort (severity class 2) in comparison to the Peruvian cohort (severity class 1), indicating more severe illness in the Kenyan TB patients, which could partly explain the differences in the methylated CpG sites between the two cohorts. To better predict TB treatment outcomes, further studies could include drug resistant TB in the cohorts and apply more strict clinical outcomes. Diagnosing TB and monitoring treatment via oral swabs would be clinically useful in limited-resource settings. Previous studies of TB biosignatures and TB treatment monitoring signatures have focused on transcriptomic signatures in the blood compartment^39–42^. In our study, we identified inherent differences in the DNAm of the PBMCs between patients at baseline and after treatment. However, we could not identify any significant DMCs using a strict cutoff of MMD ≥ 0.2 and accounting for the significant difference of cell proportions. We hypothesize that the difference in cell proportions contribute to the DNAm differences observed. Future studies could be designed to better investigate contribution of DNAm alterations by cell types in both blood and mucosa. We hypothesize that exposure to *M. tuberculosis* induces epigenetic changes in buccal cells, reflected as a changed DNAm pattern in samples collected from the oral mucosa. The DNAm pattern of active TB observed when comparing TB patients and controls was enriched in several other infectious disease pathways. Notably, many of the enriched infectious diseases pathways are caused by facultative intracellular bacteria (*Yersinia pestis*, *Shigella* and *Salmonella*) such as *M. tuberculosis.* We identified enrichment in the neurotrophin signaling pathway which we previously reported as an important pathway in regulating BCG vaccination responsiveness^43^. There was a difference in the DNAm signature of buccal cells between TB0 and HC which was not as clear among the treated TB patients (TB1-TB6). This is in line with previous knowledge suggesting that the most important clinical changes for drug-susceptible TB are seen in the first weeks of TB treatment, with reduced bacterial burden and transmissibility^44^. We observed differences in the cell proportions of epithelial cells and neutrophils over the treatment course with a rapid decrease of neutrophils after one month of treatment. Increasing the resolution of the findings with more frequent sampling of buccal swab samples could reveal when the most critical DNAm changes occur. It would also be valuable to study the DNAm changes of the buccal mucosa in TB patients for a longer time (one year or more) to follow the changes even after completed treatment. One of the limitations of the study is that we have not included healthy controls and TB-exposed in the Kenyan cohort. Moreover, we have not included other lung diseases including sarcoidosis, to differentiate the DNAm alterations we see in the buccal mucosa of TB patients with changes induced by other pulmonary diseases^45^. Diagnosing TB and monitoring treatment via buccal swabs would be clinically useful in limited-resource settings. We suggest that TB-associated DNA methylation alterations, identified with a mouth swab sample, could be used to monitor treatment of TB. DNAm can be analyzed with methylation-specific qPCR and could be aligned with existing PCR protocols if the number of addressable sites is reduced. The results suggest the possible use of DNAm-based diagnostic and prognostic tools of TB in the future.

While we applied batch-correction methods to minimize technical variability, the possibility of false positives remains. Although key pathways like 'bacterial invasion of epithelial cells’ were consistently observed in different batch-correction methods, some findings could still be influenced by technical noise. To address these limitations, we employed a machine learning approach to validate our results, where a set of DMCs demonstrated high predictive performance in determining symptom scores across multiple cohorts and external validation sets. Despite the limitations of this pilot study’s sample size, the relevance of the identified CpG sites was confirmed across independent datasets. Our results, particularly the validation of CpG sites across multiple cohorts, complement approaches such as those by Lyu et al.^46^ and Peng et al.^47^ by highlighting the consistency of methylation signatures in diverse populations and supporting the development of TB diagnostics across various datasets. Our findings suggest that DNAm signatures can be a valuable tool in TB diagnosis, but it is essential to carefully consider these limitations to ensure accurate predictions and further validation is necessary to ensure robust biological conclusions. Future studies will expand these findings by including larger populations and additional lung diseases to further validate the generalizability of the model.

## Methods

### Ethics

All research was performed in accordance with relevant guidelines and regulations in accordance with the Declaration of Helsinki. Informed consent was obtained from all participants. For the Peruvian cohort, ethical approval was obtained from the Universidad Peruana Cayetano Heredia (UPCH) Institutional Review Board (#209,390). This study is registered in the PRISA repository of the Peruvian National Health Research Registry (#EI00003140). Approval was also obtained from the local health network (Dirección de Redes Integradas de Salud—DIRIS Lima Centro) for recruitment in the healthcare centers where patients and contacts were enrolled. For the Kenyan cohort, ethical approval was obtained from MTRH/MU-Institutional Research and Ethics Committee (IREC) (#0004260). Ethical approval for analyses of samples in Sweden was obtained from the Swedish Ethical Review Authority (#2024-03200-01).

### Study design

We performed a longitudinal pilot study of patients with TB (*n* = 10), TB-exposed household- and occupational contacts (*n* = 10) and healthy controls (*n* = 10). Patients were above 18 years of age and diagnosed with drug-sensitive TB using GeneXpert MTB/RIF Ultra of sputum samples according to clinical routine, maximum 2 weeks prior to inclusion. Patients, TB-exposed and healthy controls donated buccal swab samples at baseline and at follow up after one, two and six months. We included a validation cohort of patients (*n* = 41) in Kenya at the Moi Teaching and Referral Hospital in Eldoret, Kenya. The subjects were diagnosed with TB via sputum GeneXpert MTB/RIF, urine-lipoarabinomannan, radiology or sputum smear microscopy according to the clinical routine. Oral swabs and 20 ml of peripheral blood were collected at three occasions during a six-month period in the Kenyan cohort (at treatment start, after 2 months and after 6 months of TB treatment). The subjects answered a questionnaire at inclusion, at 2 months and at 6 months. Patients with** s**evere illnesses other than TB or systemic immunosuppression were excluded from the study. The baseline samples collected within these studies have been previously used in a published study^29^, the longitudinal analyses of follow-up samples presented here are novel.

### Oral swab samples

Oral swabs (OmniSwab, Whatman Biosciences; FLOQswabs, ref 447,943; Copan Diagnostics, ref 520CS01) were used for the collection of buccal mucosal cells. Oral swab sampling is non-invasive. The buccal swab sampling was performed rubbing the buccal swab up and down on the inside of the cheek 10 times. Two oral swabs, one per cheek, were collected from each subject for DNA isolation. DNA was isolated from the oral swabs using QIAamp DNA mini kit (Qiagen). In Peru, an additional oral swab was collected and run on GeneXpert MTB/RIF Ultra.

### Interferon-gamma release assay

Venous blood was collected through venipuncture in heparin tubes. From the collected venous blood, 4 ml was used for IGRA using QuantiFERON-TB Gold (Qiagen) according to manufacturer’s instructions.

### Peripheral blood mononuclear cells isolation

In Kenya, peripheral blood mononuclear cells (PBMCs) were isolated from the remaining collected venous blood. The blood from each subject was poured into 50 ml tubes and carefully diluted with a Dulbecco’s Phosphate Buffered Saline (D-PBS) + 2% FBS solution in a 1:1 ratio. 15 ml Lymphoprep (Alere Technologies) was transferred into a SepMate tube (StemCell Technologies). The diluted sample was subsequently added to the Lymphoprep-filled tube by carefully pipetting down the side of the tube and later the SepMate tube was centrifuged at 1 200 rcf for 10 min at room temperature. After centrifugation, the plasma layer was removed without disturbing the peripheral blood mononuclear cell band. The PBMCs were poured off into a sterile 50 ml tube filled with some D-PBS and the tube was filled with cold D-PBS until a total volume of 50 ml and centrifuged at 300 rcf, 10 min in 4 °C. The supernatant was removed, and the pellet gently resuspended in D-PBS, using a transfer pipette. The sample was then filled up to 50 ml with cold D-PBS and centrifuged 220 rcf for 5 min in 4 °C. DNA from the PBMCs was extracted using QIAamp DNA mini kit (Qiagen).

### DNAm sequencing and analyses

DNA was prepared from the samples at the Laboratorios de Investigación y Desarrollo, Universidad Peruana Cayetano Heredia, Lima, Peru, and the Laboratory facilities of the AMPATH partnership at the Moi Teaching and Referral Hospital in Eldoret, Kenya. Prepared DNA was shipped to Sweden and analyzed with Illumina Infinium MethylationEPIC BeadChip 850 K microarray as per the manufacturer´s instructions. The methylation profiles from the 850 K BeadChip array were analyzed using the raw intensity (IDAT) files in R (v4.0.2). In short, the data were pre-processed in several steps including removal of probes that have failed in multiple samples and/or overlapping SNPs followed by imputation using the *ChAMP* (v2.19.3) package in R. BMIQ Normalization was performed on the filtered data. Singular value decomposition (SVD) analysis was performed, and the data was batch corrected for slide and array if significant contribution was identified using *ChAMP* (v2.19.3) The SVD results before and after batch correction for all datasets are presented in supplementary Figs. 7–10. The cell type proportions in the buccal swabs were analyzed using HEpiDISH package which uses houseman algorithm for the immune cell types. For differential methylation analysis, we used linear model in R package limma. In the DMC identification models we included the cell proportions of cells significantly different between groups and the significant covariates (Age, Sex, BMI) presented in the SVD after correction (with one exception for the longitudinal analysis of TB0 and TB6 in the pilot cohort as mentioned in the result). In the case of longitudinal data analysis, the individual effect (samples from the same individual tend to be correlated) was modeled as a random effect using the function *duplicateCorrelation*. For details, the DNAm analysis pipeline used is available at https://github.com/Lerm-Lab/TB_Treatment_DNAm_Buccal_Swabs.

### Disease pathway analysis

To identify the disease module genes that interact with each other based on the identified DMGs (*n* = 242 entrez gene IDs), we have used Disease Module Detection (DIAMOnD) algorithm that is part of MODifieR package v. 0.1.3 and STRING protein protein interaction network. In STRING network, we used high confidence with threshold of 700. In the DIAMOnD algorithm, we set parameters: cut-off = 0.05, number of connectors = 200, and seed weight = 10. The resulting disease module genes *n* = 416 were used to find the KEGG pathway enrichment with R clusterProfiler package v.4.10.1. The results were plotted using dotplot using enrichplot package v.1.14.2. Furthermore, an online database of protein protein interaction network analysis, STRING (version 12, https://string-db.org/cgi/input?sessionId=bVXztLSEyM40) and Network of functional couplings (FunCoup: https://funcoup.org/search/) were used to identify the KEGG enriched pathways by giving the DMGs as an input and building a network around those input genes to find candidate biological pathways.

### Statistical analyses

All differences with an FDR corrected *p*-value < 0.05 were considered significant if not otherwise stated. All analyses were performed in R (v4.0.2) with the mentioned packages. The demographic data was analyzed in SPSS, using Kruskal Wallis analysis for comparison between patients, exposed and healthy controls in the Peruvian cohort. For significant results, exact significance was calculated between two groups using Mann–Whitney U-test. All comparisons of cell proportions were performed using 2-way ANOVA and Tukey´s multiple comparisons test.

### Machine learning feature and model selection

To identify the most relevant CpG sites for the prediction of symptom score, we performed feature selection using elastic net regression on DNAm data from the Kenyan cohort. The 468 DMCs identified between TB0 patients and HC from the Peruvian cohort were used as the starting feature set. A five-fold cross-validation (CV) scheme was employed to partition the data, maintaining individual-level grouping across folds to prevent data leakage by ensuring that longitudinal samples from the same individual remained in the same training or test set. For each fold, we applied elastic net with an L1/L2 regularization ratio of 0.5 to select a subset of CpG sites with non-zero coefficients, representing features contributing to the model’s predictive power. Performance was measured using R-squared (R^2^), Pearson correlation, mean absolute error (MAE), and mean squared error (MSE). CpG sites selected in each fold were aggregated, with those that appeared in every fold *being considered as robust predictors for further analysis.* Next, we trained and evaluated eight different linear and non-linear regression models (linear, ridge, lasso, elastic net, random forest (RF) regressor, support vector regressor (SVR), XGBoost, and k-nearest neighbors (kNN) models) using the previously selected CpG sites. For each model, if applicable, up to seven hyperparameters were optimized using a grid search with five-fold CV, assessing the performance using the average MSE. After selecting the best hyperparameters for each model, we again trained and evaluated their performance on the Kenyan cohort, split into training and test sets (80:20 ratio) with individual-level grouping. The elastic net regressor with intercept, alpha = 1.63e-3, L1 ratio = 0.1, maximum iterations = 1e3, and tolerance = 1e-4 was chosen for further training and validation using independent cohorts.

### Machine learning model training and validation

To validate and determine the specificity of the selected CpG sites and regression model, we trained and evaluated its performance using two publicly available cohorts of buccal swab DNA methylation samples from healthy individuals measured using the Illumina MethylationEPIC array (cohort A: GSE147058, *n* = 96; cohort B: GSE137841, *n* = 250). The elastic net model was trained using the selected hyperparameters on TB patients from the Kenyan cohort training set and healthy individuals from cohort A (total *n* = 162), with sample weights proportional to the size of each cohort to account for sample size imbalance. Samples from healthy cohorts were assigned a symptom score of 0. To improve the distributional properties of the target variable, symptom scores were scaled between 0 and 1, before applying a logit transformation, adding epsilon = 1e-2 to the scores to ensure numerical stability. Model coefficients were forced to be positive to ensure that the relationship between features and symptom scores remained biologically interpretable. After training, nine out of 13 CpG sites had non-zero coefficients. The validation set consisted in the TB patients from the Kenyan cohort test set and the Peruvian cohort (TB baseline), and the healthy individuals from cohort B (total *n* = 276). The code used for feature selection, model selection, hyperparameter optimization, training and validation of symptom score prediction machine learning models from DNA methylation data is available in Zenodo with the identifier https://doi.org/10.5281/zenodo.13929188.

## Supplementary Information

Supplementary Information 1.

Supplementary Information 2.

Supplementary Information 3.

## Data Availability

The datasets generated during and analyzed during the current study are not publicly available due to ethical dilemmas in traceability of DNA methylation data, but processed data that is pseudonymized and depleted of genetic variant information (beta matrixes with Illumina probe IDs and beta values) are available from the corresponding author on reasonable request. The external cohorts GSE147058 and GSE137841 are available for download in the Gene Expression Omnibus (GEO) repository. The trained regressor models for the prediction of symptom score, optimal hyperparameters, and selected features are available at https://figshare.com/projects/610956_buccal_swab_TB_symptom_score_regressor/222726.
