# Supplementary material for: A DNA methylation signature identified in the buccal mucosa reflecting active tuberculosis is changing during tuberculosis treatment
Source: Sci Rep. 2024 Nov 28;14:29552. doi: 10.1038/s41598-024-80570-4 (PMC11604703; doi:10.1038/s41598-024-80570-4)
Supplement: Supplementary file 1 — Supplementary Information 1. [file 41598_2024_80570_MOESM1_ESM.pdf]

A DNA methylation signature identified in the buccal mucosa reflecting active tuberculosis is changed during tuberculosis treatment

Isabelle Öhrnberg[1†], Lovisa Karlsson[1†], Shumaila Sayyab[1], Jakob Paues[1,2], Patricia Espinoza-Lopez[3,4], Melissa Méndez-Aranda[5], Ericka Meza[4], Cesar Ugarte-Gil[3,4,6], Nicholas Kiprotich[7,8], Lameck Diero[8,9], Ronald Tonui[8,10], Maria Lerm[1*]

[1]Division of Inflammation and Infection, [2]Division of Infectious Diseases, Department of Biomedical and Clinical Sciences, Faculty of Medicine and Health Sciences, Linköping University, Linköping, Sweden, [3]Facultad de Medicina, [4]Instituto de Medicina Tropical Alexander Von Humboldt, [5]Laboratorios de Investigación y Desarrollo, Facultad de Ciencias e Ingeniería, Universidad Peruana Cayetano Heredia, Lima, Peru, [6]Department of Epidemiology, School of Public and Population Health, University of Texas Medical Branch Galveston, Texas, USA, [7]Biochemistry and Clinical chemistry [8]AMPATH Kenya, [9]Department of Medicine, [10]Department of Pathology, Moi University Eldoret, Kenya

†) Isabelle Öhrnberg and Lovisa Karlsson contributed equally to this work

Supplementary figures

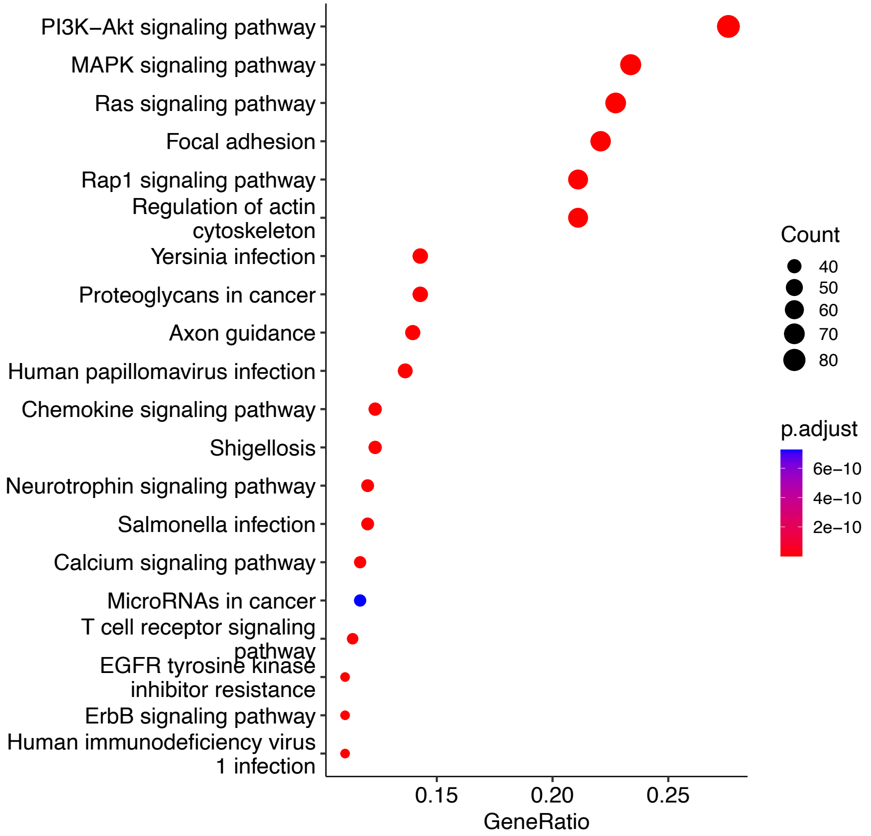

Supplementary figure 1. **KEGG enrichment analysis of differentially methylated CpG sites identified between TB patients and healthy controls.** Differentially methylated CpG sites (MMD >0.2 and p. adj <0.05) identified between active TB patients and controls in Peruvian pilot cohort were used in a disease module detection algorithm (DIAMOnD) and further a pathway enrichment analysis using KEGG. The top 20 enriched pathways are represented in the plot with GeneRatio on x-axis, number of genes in size of the dot and p. adj in colour of the dots. KEGG, Kyoto Encyclopedia of Genes and Genomes; MMD, mean methylation difference; TB, tuberculosis; p.ajd, adjusted p-value

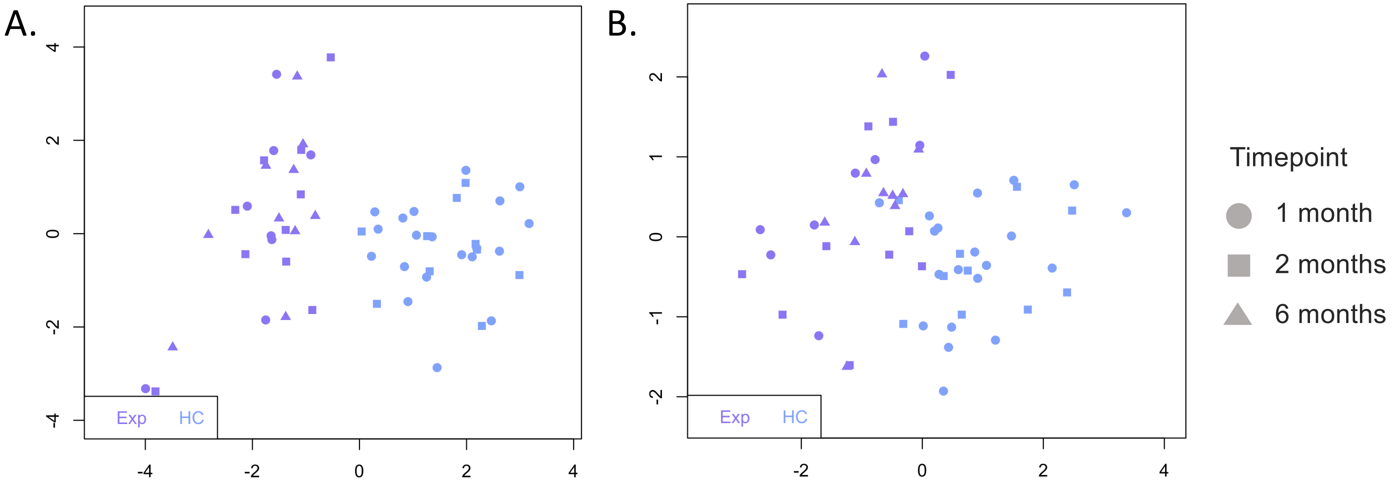

Supplementary Figure 2. **Longitudinal DNA methylation data from buccal swab samples of TB exposed and healthy controls followed over 6 months. A**. An MDS of the 1,000 of the most variable CpG sites in DNAm data from buccal swabs collected from healthy controls (HC) and TB exposed (Exp) followed 1, 2 and 6 months after inclusion. **B.** An MDS of 468 DMCs (mean methylation difference 0.2, p.adj <0.05) identified between TB patients at baseline (TB0) and healthy controls (HC0). The MDS is showing the HC followed over time with samples collected after 1 month (TB1), after 2 months (TB2) and after 6 months (TB6). DNAm, DNA methylation; TB, tuberculosis; MDS, multidimensional scaling; DMCs, differentially methylated CpG sites; MMD, mean methylation difference; p.ajd, adjusted p-value; IGRA, interferon-gamma release assay

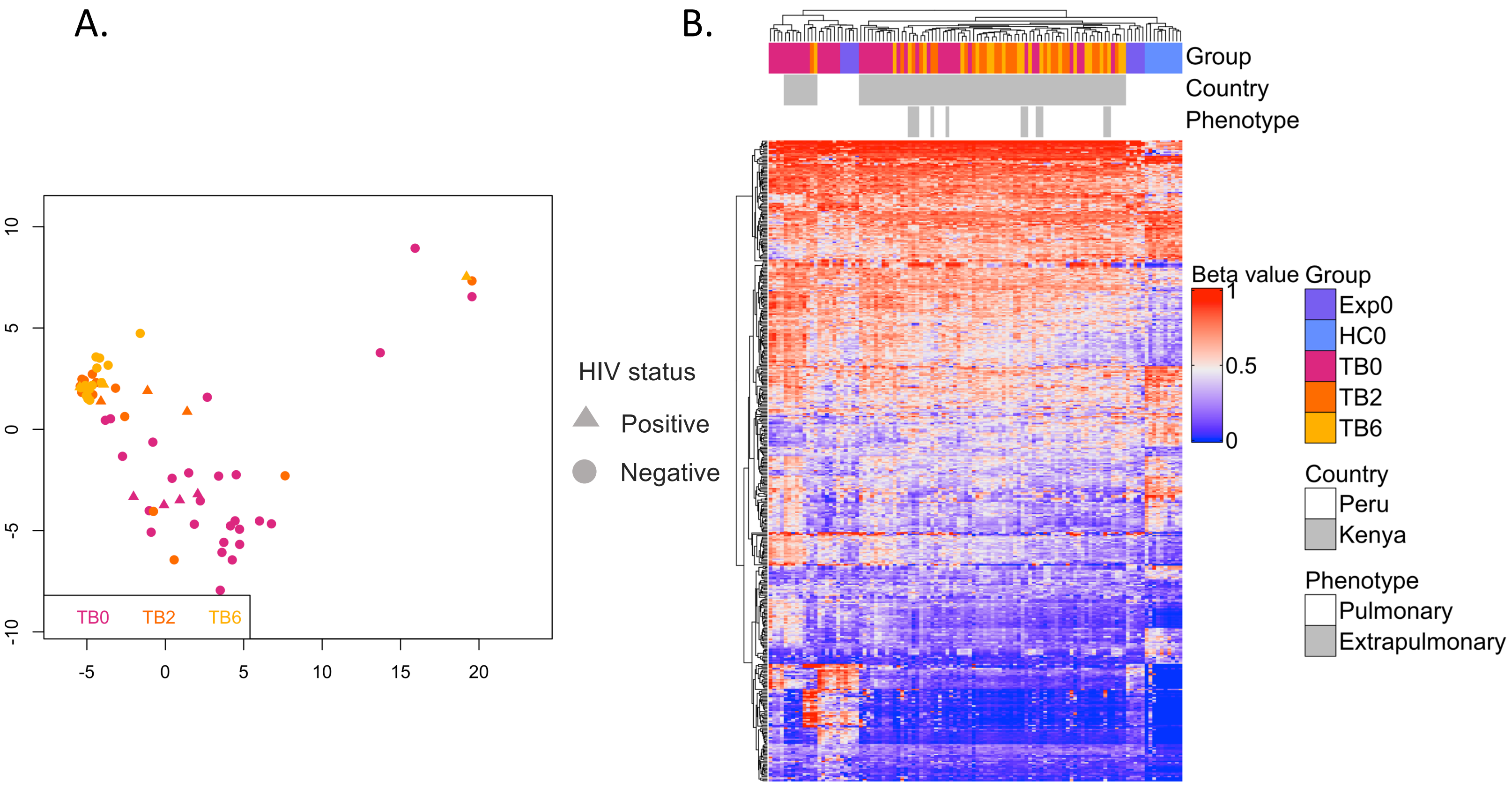

Supplementary Figure 3. **DNA methylation patterns from buccal swab samples of tuberculosis patients followed during treatmernt with HIV coinfection and extrapulmonaty TB disease phenotype. A.** Multidimentional scaling (MDS) plot of the 1,000. most variable CpG sites in the validation cohort showing TB patients at baseline (TB0), after 2 and 6 months of treatment (TB2 and TB6). HIV positive patients indicated with triangle. **B**. Heatmap plot of 468 DMCs (mean methylation difference (MMD) 0.2, p.adj >0.05) identified between TB patients at baseline (TB0) and healthy controls (HC0) in the pilot cohort. Baseline samples from the pilot cohort and TB0, TB2 and TB6 from the validation cohort included in the plot. Five extrapulmonary TB patients included in the heatmap are indicated by colour in the top bar (Phenotype). TB, tuberculosis; MDS, multidimensional scaling; DMCs, differentially methylated CpG sites; MMD, mean methylation difference; p.ajd, adjusted p-value

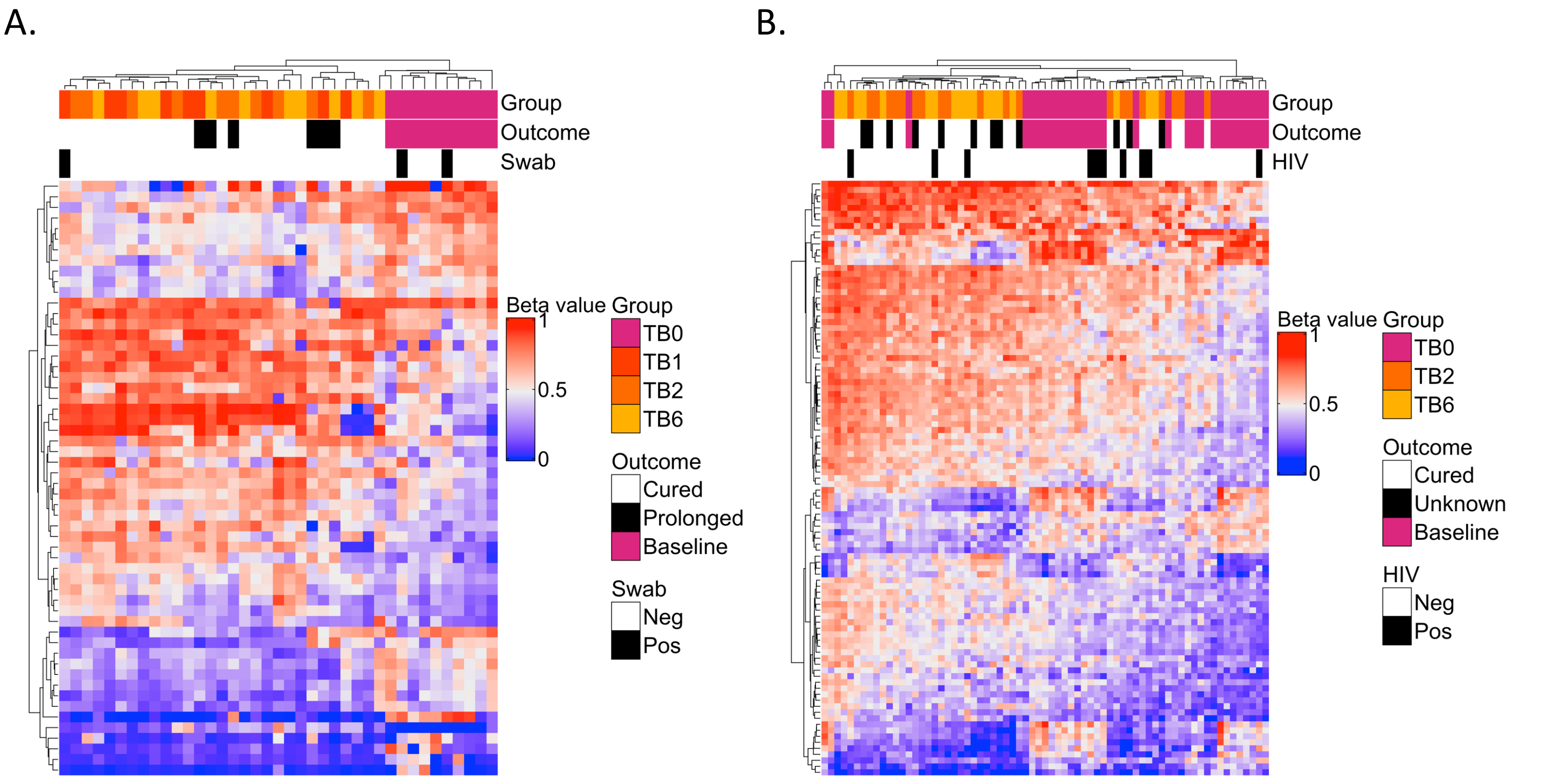

Supplementary figure 4. **DNAm changes in the buccal mucosa of TB patients undergoing treatment with sampling at baseline (TB0) and after one (TB1), two (TB2) and six (TB6) months. A.** A heatmap of 56 CpG sites differentially methylated (mean methylation difference >0.2, adj p value <0.05) CpG sites (DMCs) between TB0 and TB6 in pilot cohort. Treatment outcome (Outcome) and GeneXpert positive swab samples (Swab) are indicated in the top bar. **B**. A heatmap of beta values from 99 DMCs between TB0 and TB6 in the validation cohort indicating treatment outcome and HIV coinfection status in the top bar. DNAm, DNA methylation; TB, TB patients; MDS, multidimensional scaling; DMCs, differentially methylated CpG sites; MMD, mean methylation difference; p.ajd, adjusted p-value.

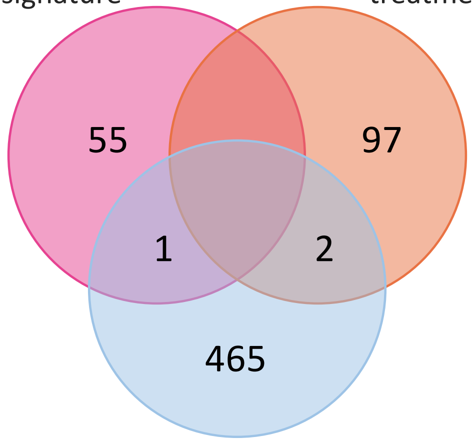

Supplementary Figure 5. **A Venn diagram of overlappning DMCs.** Overlap in DNA methylation signatures identified in buccal mucosal samples in TB patients during TB treatment and between TB patients and healthy controls. The Pilot TB disease signature consisting of 468 differentially methylated CpG sites (DMCs) (mean methylation difference >0.2 and adj. p <0.05) was identified by comparing TB patients and healthy controls from Peru at baseline. The pilot treatment signature consisting of 56 DMCs was identified by comparing TB patients at baseline and after 6 months of TB treatment. The Validation treatment signature was identified by investigating an indpendent cohort from Kenya and by comparing TB patients at baseline and TB patients after 6 months of treatment.

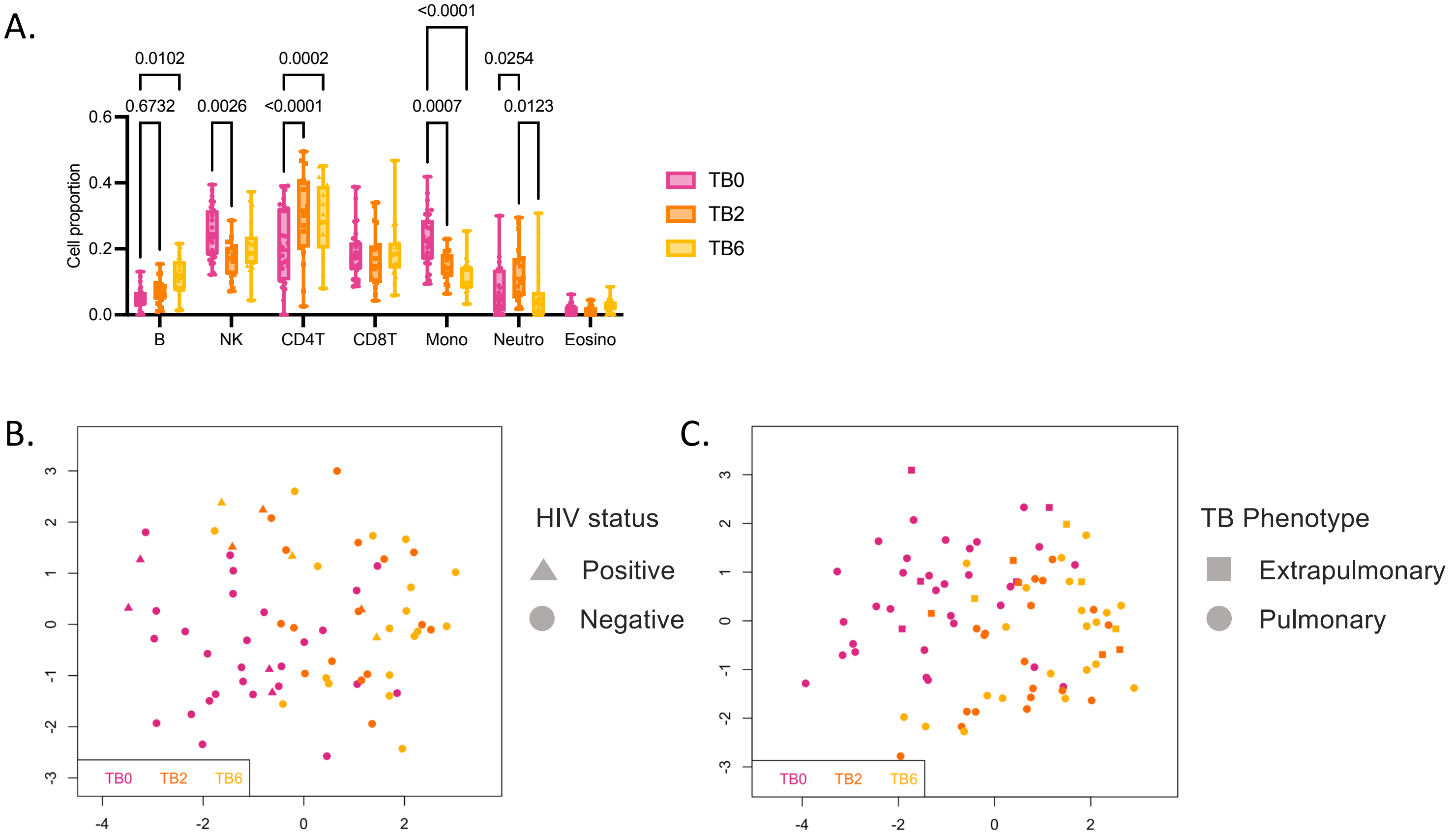

Supplementary Figure 6. **DNA methylation data from peripheral blood mononuclear cell samples of Kenyan validation cohort.**
A. Cell type deconvolution of PBMC samples. The statistical analysis was performed with with two-way ANOVA and Tukey multipel comparisons test **B.** MDS plot of the 1,000 most variable CpG sites in PBMCs collected from pulmonary TB patients in Kenyan validation cohort. TB patients at baseline (TB0) in pink, after two months of treatment (TB2) in orange and after six months of treatment (TB6) in yellow. HIV coinfected patients indicated with triangle. **C.** MDS of the 1,000 most variable CpG sites in PBMC collected from pulmonary and extrapulmonary TB patients at TB0 in pink, TB2 in orange and TB6 in yellow. Extrapulmonary TB phenotype indicated with square and pulmonary with circle. PBMC, peripehral blood mononuclear cells; TB, tuberculosis; MDS, multidimensional scaling

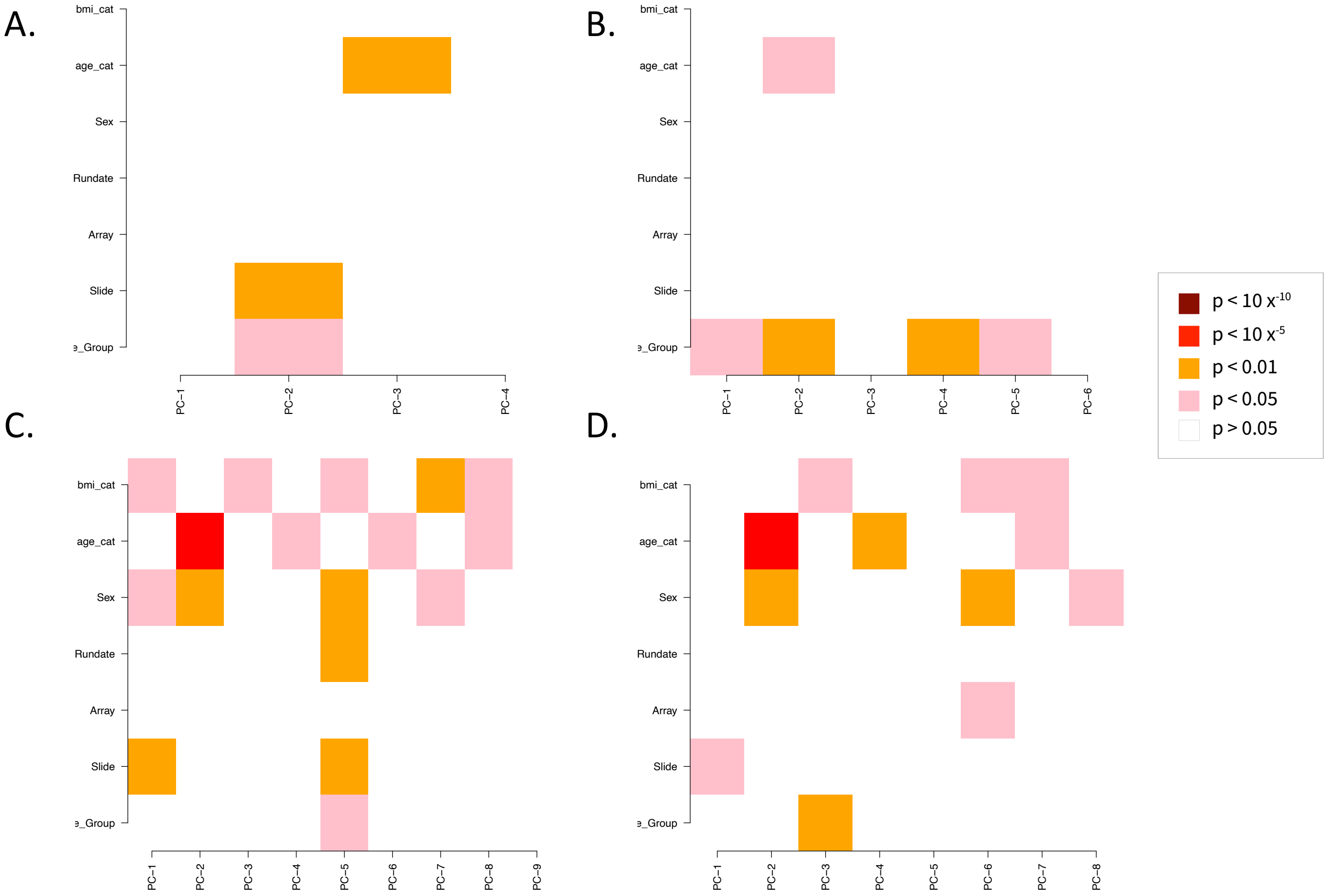

Supplementary Figure 7. **Singular value decomposition (SVD) of TB patients, TB exposed and healthy controls in Peruvian pilot cohort.** SVD of DNAm data from buccal swab samples from TB patients, TB exposed and healthy controls before (A) and after (B) batch correction. **C.** SVD of DNAm data from buccal swabs of TB patients undergoing treatment including samples at baseline, 1, 2 and 6 months before correction. **D.** After correction.

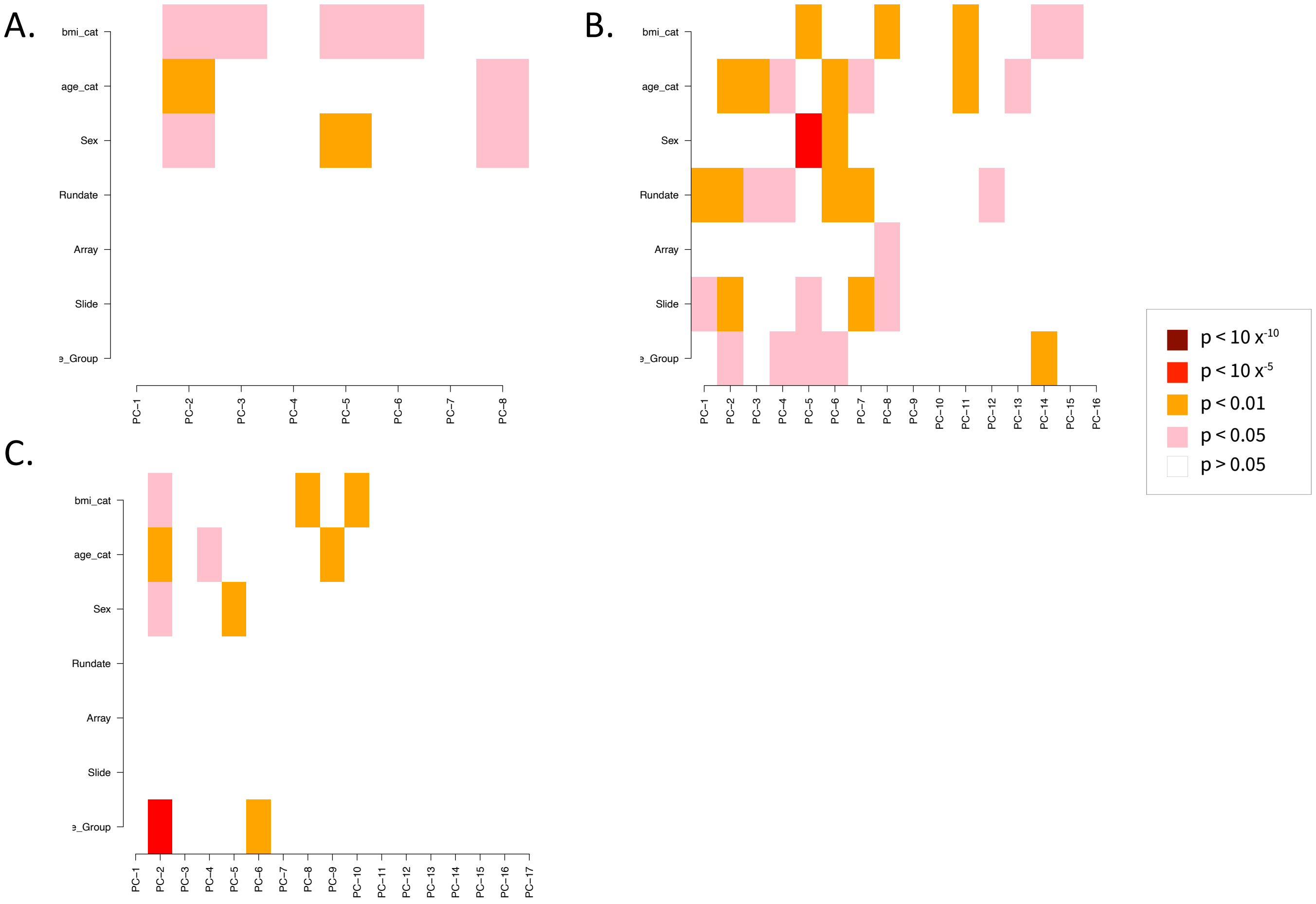

Supplementary Figure 8**. Single value decomposition (SVD) of TB patients during treatment in Peruvian pilot cohort. A.** SVD of DNAm data from buccal swab samples from TB patients after 1, 2 and 6 months of TB treatment. **B.** SVD of DNAm data from buccal swab samples from healthy controls and TB exposed individuals with samples collected 1,2 and 6 months after inclusion before correction. **C.** After correction.

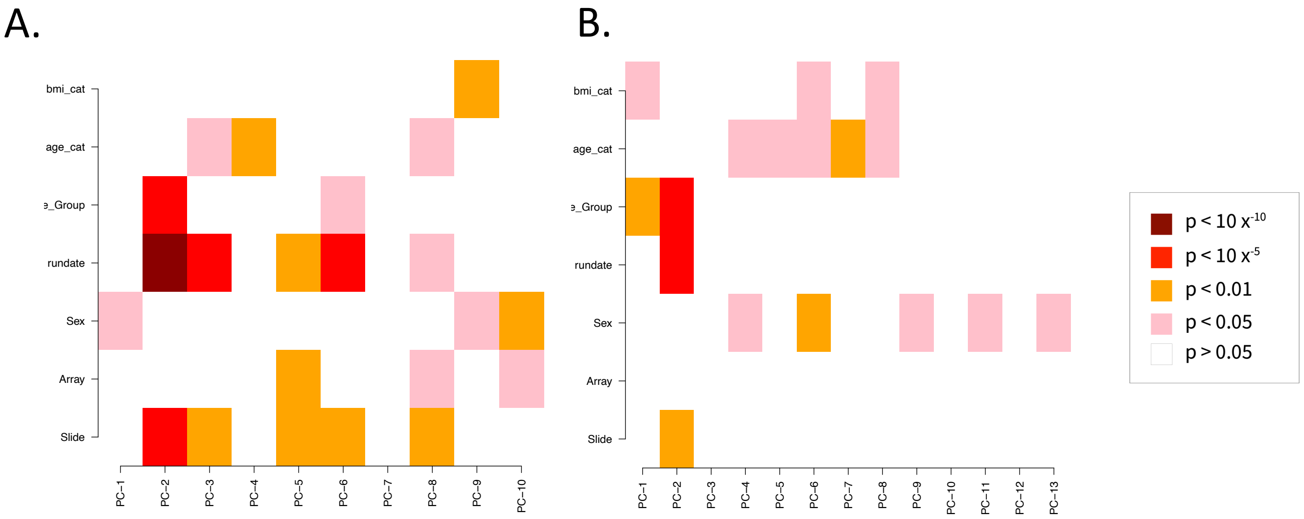

Supplementary Figure 9. **Singular value decomposition (SVD) of Kenyan validation cohort**. **A.** SVD of DNA methylation data from buccal swabs of the validation cohort before batch correction. **B.** SVD after batch correction.

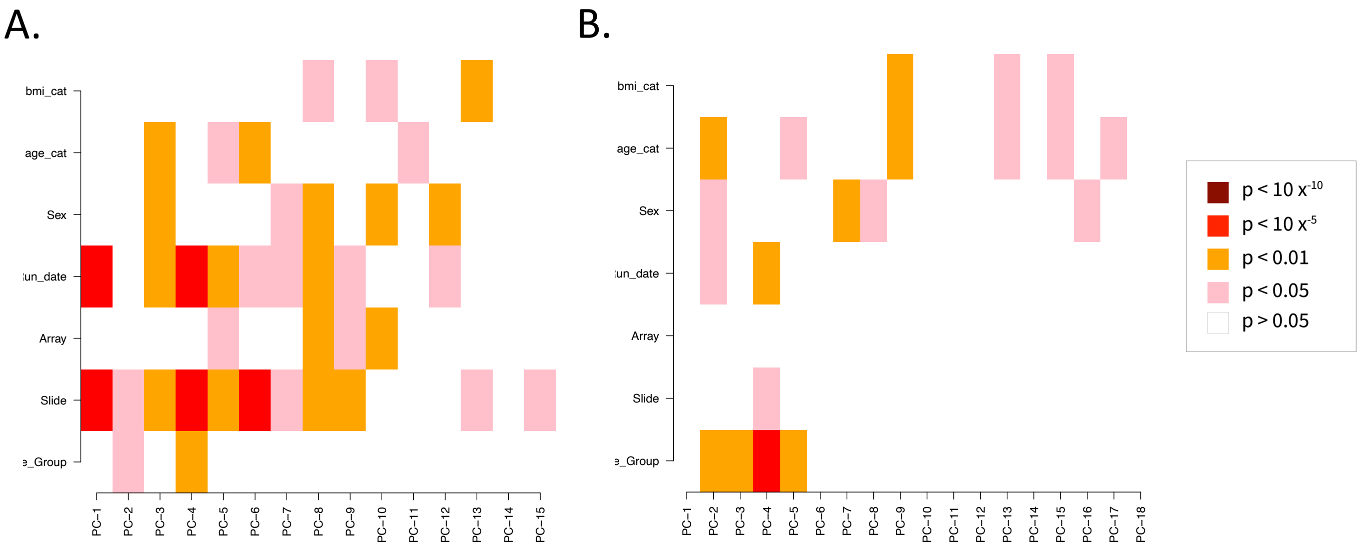

Supplementary Figure 10. **A.** Sigular value decomposition (SVD) of DNA methylation data from peripheral blood mononuclear cell samples collected from Kenyan validation cohort before batch correction. **B.** SVD after batch correction.
