# Supplementary material for: A DNA methylation signature identified in the buccal mucosa reflecting active tuberculosis is changing during tuberculosis treatment
Source: Sci Rep. 2024 Nov 28;14:29552. doi: 10.1038/s41598-024-80570-4 (PMC11604703; doi:10.1038/s41598-024-80570-4)
Supplement: Supplementary file 2 — Supplementary Information 2. [file 41598_2024_80570_MOESM2_ESM.pdf]

A DNA methylation signature identified in the buccal mucosa reflecting active tuberculosis is changed during tuberculosis treatment

Isabelle Öhrnberg[1†], Lovisa Karlsson[1†], Shumaila Sayyab[1], Jakob Paues[1,2], Patricia Espinoza-Lopez[3,4], Melissa Méndez-Aranda[5], Ericka Meza[4], Cesar Ugarte-Gil[3,4,6], Nicholas Kiprotich[7,8], Lameck Diero[8,9], Ronald Tonui[8,10], Maria Lerm[1*]

[1]Division of Inflammation and Infection, [2]Division of Infectious Diseases, Department of Biomedical and Clinical Sciences, Faculty of Medicine and Health Sciences, Linköping University, Linköping, Sweden, [3]Facultad de Medicina, [4]Instituto de Medicina Tropical Alexander Von Humboldt, [5]Laboratorios de Investigación y Desarrollo, Facultad de Ciencias e Ingeniería, Universidad Peruana Cayetano Heredia, Lima, Peru, [6]Department of Epidemiology, School of Public and Population Health, University of Texas Medical Branch Galveston, Texas, USA, [7]Biochemistry and Clinical chemistry [8]AMPATH Kenya, [9]Department of Medicine, [10]Department of Pathology, Moi University Eldoret, Kenya

†) Isabelle Öhrnberg and Lovisa Karlsson contributed equally to this work

Supplementary list 1. Differentially methylated CpG sites (MMD >0.2 and p. adj <0.05) identified between active TB patients and controls in Peruvian pilot cohort were used in a disease module detection algorithm (DIAMOnD) and further a pathway enrichment analysis using KEGG. All significantly enriched pathway are listed.

ID	Description	GeneRatio	p.adjust
hsa04151	PI3K-Akt signaling pathway	85/308	1.15632993171775e-46
hsa04010	MAPK signaling pathway	72/308	5.10420914334416e-40
hsa04014	Ras signaling pathway	70/308	4.82679363921646e-45
hsa04510	Focal adhesion	68/308	1.94844161830656e-47
hsa04015	Rap1 signaling pathway	65/308	5.41774865687103e-43
hsa04810	Regulation of actin cytoskeleton	65/308	1.36380257372309e-40
hsa05135	Yersinia infection	44/308	1.68917246562465e-29
hsa05205	Proteoglycans in cancer	44/308	5.49763476513073e-22
hsa04360	Axon guidance	43/308	6.36287241080503e-23
hsa05165	Human papillomavirus infection	42/308	1.30309086146068e-12
hsa04062	Chemokine signaling pathway	38/308	1.25199103353272e-17
hsa05131	Shigellosis	38/308	5.00778346117786e-14
hsa04722	Neurotrophin signaling pathway	37/308	3.00759632281405e-24
hsa05132	Salmonella infection	37/308	3.2335969120482e-13
hsa04020	Calcium signaling pathway	36/308	2.2014805627574e-12
hsa05206	MicroRNAs in cancer	36/308	7.25117389777283e-10
hsa04660	T cell receptor signaling pathway	35/308	7.91543743197193e-22
hsa01521	EGFR tyrosine kinase inhibitor resistance	34/308	1.52221300095771e-27
hsa04012	ErbB signaling pathway	34/308	2.59728209329829e-26
hsa05170	Human immunodeficiency virus 1 infection	34/308	3.35194472189026e-13
hsa05163	Human cytomegalovirus infection	34/308	1.89179292211814e-12
hsa05226	Gastric cancer	33/308	6.75300661010171e-17
hsa05224	Breast cancer	32/308	3.51397290952783e-16
hsa04072	Phospholipase D signaling pathway	32/308	4.17664681172176e-16
hsa05218	Melanoma	31/308	3.89380885838785e-25
hsa04666	Fc gamma R-mediated phagocytosis	31/308	9.70027613602581e-21
hsa05231	Choline metabolism in cancer	31/308	1.28344439336814e-20
hsa04935	Growth hormone synthesis, secretion and action	31/308	1.0253494242708e-17
hsa04910	Insulin signaling pathway	31/308	3.51397290952783e-16
hsa05130	Pathogenic Escherichia coli infection	31/308	1.29331649370283e-11
hsa05208	Chemical carcinogenesis - reactive oxygen species	31/308	2.06130879253116e-10
hsa04650	Natural killer cell mediated cytotoxicity	30/308	1.49366180242902e-15
hsa05167	Kaposi sarcoma-associated herpesvirus infection	30/308	2.75300877328688e-11
hsa05100	Bacterial invasion of epithelial cells	28/308	1.75762956908535e-20
hsa01522	Endocrine resistance	28/308	2.17374841306667e-17
hsa05161	Hepatitis B	28/308	8.49175971584006e-12
hsa04926	Relaxin signaling pathway	27/308	2.22916689743612e-13
hsa04380	Osteoclast differentiation	27/308	1.91174677543696e-12
hsa05207	Chemical carcinogenesis - receptor activation	27/308	2.1560709696495e-08
hsa05417	Lipid and atherosclerosis	27/308	2.35523182619437e-08

hsa05211	Renal cell carcinoma	26/308	1.78608129409408e-19
hsa04613	Neutrophil extracellular trap formation	26/308	8.75076387093876e-09
hsa05166	Human T-cell leukemia virus 1 infection	26/308	1.77218152959365e-07
hsa04024	cAMP signaling pathway	26/308	2.30127047537319e-07
hsa04662	B cell receptor signaling pathway	25/308	2.47096822151896e-15
hsa04933	AGE-RAGE signaling pathway in diabetic complications	25/308	3.00793798517917e-14
hsa04664	Fc epsilon RI signaling pathway	24/308	2.81179791211463e-17
hsa04917	Prolactin signaling pathway	24/308	5.80534308721971e-17
hsa05210	Colorectal cancer	24/308	8.14851066746316e-15
hsa05235	PD-L1 expression and PD-1 checkpoint pathway in cancer	24/308	1.79126968179211e-14
hsa05215	Prostate cancer	24/308	1.30038903857265e-13
hsa04670	Leukocyte transendothelial migration	24/308	5.58490373770724e-12
hsa05418	Fluid shear stress and atherosclerosis	24/308	3.81010289544489e-10
hsa04140	Autophagy - animal	24/308	1.39554355441419e-08
hsa05010	Alzheimer disease	24/308	0.00943593473348421
hsa05214	Glioma	23/308	3.54187664598409e-15
hsa04150	mTOR signaling pathway	23/308	1.81000267156629e-08
hsa05230	Central carbon metabolism in cancer	22/308	8.46811533863156e-15
hsa05220	Chronic myeloid leukemia	22/308	5.1106720202561e-14
hsa04520	Adherens junction	22/308	2.94930094582342e-12
hsa04668	TNF signaling pathway	22/308	4.38031746853701e-10
hsa04071	Sphingolipid signaling pathway	22/308	7.03126503410542e-10
hsa04068	FoxO signaling pathway	22/308	3.71083408157919e-09
hsa04932	Non-alcoholic fatty liver disease	22/308	7.26797590996929e-08
hsa05225	Hepatocellular carcinoma	22/308	2.94920542347234e-07
hsa05203	Viral carcinogenesis	22/308	6.80448617632538e-06
hsa05022	Pathways of neurodegeneration - multiple diseases	22/308	0.18978424981308
hsa04370	VEGF signaling pathway	21/308	2.51589847943718e-15
hsa04931	Insulin resistance	21/308	5.18341766336943e-10
hsa04611	Platelet activation	21/308	6.45985766965227e-09
hsa04210	Apoptosis	21/308	2.78769708366302e-08
hsa05169	Epstein-Barr virus infection	21/308	1.98506398644567e-05
hsa05415	Diabetic cardiomyopathy	21/308	2.22631773532849e-05
hsa04144	Endocytosis	21/308	0.00041764352690289
hsa05212	Pancreatic cancer	20/308	5.1266515569579e-12
hsa05222	Small cell lung cancer	20/308	1.94267609154283e-10
hsa04625	C-type lectin receptor signaling pathway	20/308	1.71669983651337e-09
hsa04659	Th17 cell differentiation	20/308	2.85833966355848e-09
hsa05145	Toxoplasmosis	20/308	4.53035748744337e-09
hsa04148	Efferocytosis	20/308	1.26545192910577e-06
hsa05160	Hepatitis C	20/308	1.68919722234753e-06

hsa05152	Tuberculosis	20/308	1.14516393204828e-05
hsa04066	HIF-1 signaling pathway	19/308	1.94782510871124e-08
hsa04915	Estrogen signaling pathway	19/308	9.22411008887775e-07
hsa04218	Cellular senescence	19/308	5.75821443820785e-06
hsa04630	JAK-STAT signaling pathway	19/308	1.52469163909484e-05
hsa05223	Non-small cell lung cancer	18/308	1.67712708925235e-10
hsa04658	Th1 and Th2 cell differentiation	18/308	7.65144522347912e-09
hsa05146	Amoebiasis	18/308	4.45983567018988e-08
hsa04620	Toll-like receptor signaling pathway	18/308	1.07857285557854e-07
hsa05171	Coronavirus disease - COVID-19	18/308	0.00326322257174331
hsa05221	Acute myeloid leukemia	17/308	4.38031746853701e-10
hsa04512	ECM-receptor interaction	17/308	2.85522277851357e-08
hsa04912	GnRH signaling pathway	17/308	5.5788249875776e-08
hsa05142	Chagas disease	17/308	2.54605180842845e-07
hsa04550	Signaling pathways regulating pluripotency of stem cells	17/308	2.50754369975954e-05
hsa04530	Tight junction	17/308	0.000199548072692529
hsa05164	Influenza A	17/308	0.00022883384902732
hsa05020	Prion disease	17/308	0.0305468952147918
hsa05162	Measles	16/308	6.13128657180872e-05
hsa05034	Alcoholism	16/308	0.00179438073335788
hsa04930	Type II diabetes mellitus	15/308	1.33717198335543e-10
hsa05213	Endometrial cancer	15/308	3.88698468307025e-09
hsa04540	Gap junction	15/308	1.62764450922355e-06
hsa04914	Progesterone-mediated oocyte maturation	15/308	1.70484003058389e-05
hsa04919	Thyroid hormone signaling pathway	15/308	5.05003824360643e-05
hsa04820	Cytoskeleton in muscle cells	15/308	0.0284220064333507
hsa04920	Adipocytokine signaling pathway	14/308	2.96887622613689e-07
hsa05120	Epithelial cell signaling in Helicobacter pylori infection	14/308	3.54179564112437e-07
hsa05133	Pertussis	14/308	1.18095869357747e-06
hsa04750	Inflammatory mediator regulation of TRP channels	14/308	1.98506398644567e-05
hsa04921	Oxytocin signaling pathway	14/308	0.00194397889620744
hsa05140	Leishmaniasis	13/308	5.96994248486519e-06
hsa05412	Arrhythmogenic right ventricular cardiomyopathy	13/308	1.98506398644567e-05
hsa04211	Longevity regulating pathway	13/308	3.18088425891427e-05
hsa05410	Hypertrophic cardiomyopathy	13/308	8.70369032026557e-05
hsa05414	Dilated cardiomyopathy	13/308	0.000159911574231215
hsa04152	AMPK signaling pathway	13/308	0.000684608249374517
hsa04936	Alcoholic liver disease	13/308	0.00305379881242509
hsa05017	Spinocerebellar ataxia	13/308	0.00305379881242509
hsa05202	Transcriptional misregulation in cancer	13/308	0.0305468952147918
hsa04064	NF-kappa B signaling pathway	12/308	0.000615374154298498

hsa04725	Cholinergic synapse	12/308	0.00136783438527749
hsa04928	Parathyroid hormone synthesis, secretion and action	12/308	0.00136783438527749
hsa04270	Vascular smooth muscle contraction	12/308	0.00480247266837948
hsa04371	Apelin signaling pathway	12/308	0.00680167454754356
hsa04022	cGMP-PKG signaling pathway	12/308	0.0242885314119625
hsa04714	Thermogenesis	12/308	0.18978424981308
hsa04213	Longevity regulating pathway - multiple species	11/308	2.05584035433715e-05
hsa04657	IL-17 signaling pathway	11/308	0.000957731777169636
hsa04916	Melanogenesis	11/308	0.00158085626218277
hsa04137	Mitophagy - animal	11/308	0.00212224871387845
hsa04723	Retrograde endocannabinoid signaling	11/308	0.0273512474435213
hsa04514	Cell adhesion molecules	11/308	0.0368354498103312
hsa04621	NOD-like receptor signaling pathway	11/308	0.110577510442625
hsa05219	Bladder cancer	10/308	2.79274902228893e-06
hsa04929	GnRH secretion	10/308	0.000174482183912537
hsa05416	Viral myocarditis	10/308	0.000285364476099354
hsa04640	Hematopoietic cell lineage	10/308	0.00454059766390379
hsa04728	Dopaminergic synapse	10/308	0.0299977815059351
hsa04310	Wnt signaling pathway	10/308	0.136499065180674
hsa05016	Huntington disease	10/308	0.828035323816674
hsa04730	Long-term depression	9/308	0.000467093292943326
hsa04070	Phosphatidylinositol signaling system	9/308	0.0133145684843156
hsa04726	Serotonergic synapse	9/308	0.0328709256471157
hsa04145	Phagosome	9/308	0.159115055656215
hsa05014	Amyotrophic lateral sclerosis	9/308	0.96823632820802
hsa05216	Thyroid cancer	8/308	7.70507632848456e-05
hsa04960	Aldosterone-regulated sodium reabsorption	8/308	9.29570016044916e-05
hsa04923	Regulation of lipolysis in adipocytes	8/308	0.00194397889620744
hsa04622	RIG-I-like receptor signaling pathway	8/308	0.00669837529976552
hsa01524	Platinum drug resistance	8/308	0.00850432476222206
hsa05323	Rheumatoid arthritis	8/308	0.029696571431958
hsa04114	Oocyte meiosis	8/308	0.18070424876283
hsa05322	Systemic lupus erythematosus	8/308	0.18070424876283
hsa04261	Adrenergic signaling in cardiomyocytes	8/308	0.262894884326332
hsa04934	Cushing syndrome	8/308	0.267848223342505
hsa04390	Hippo signaling pathway	8/308	0.279419837013711
hsa04217	Necroptosis	8/308	0.289450003355871
hsa05340	Primary immunodeficiency	7/308	0.000639913101083007
hsa04720	Long-term potentiation	7/308	0.0158701040030047
hsa04350	TGF-beta signaling pathway	7/308	0.136499065180674
hsa05012	Parkinson disease	7/308	0.947440322539008
hsa04060	Cytokine-cytokine receptor interaction	7/308	0.96823632820802
hsa05144	Malaria	6/308	0.0140888060524997

hsa00562	Inositol phosphate metabolism	6/308	0.0704582313998889
hsa04713	Circadian entrainment	6/308	0.192262627108512
hsa04724	Glutamatergic synapse	6/308	0.320426139168782
hsa05143	African trypanosomiasis	5/308	0.016174495298583
hsa04940	Type I diabetes mellitus	5/308	0.0293367139703719
hsa04973	Carbohydrate digestion and absorption	5/308	0.0583817584081621
hsa04115	p53 signaling pathway	5/308	0.190118328463184
hsa04061	Viral protein interaction with cytokine and cytokine receptor	5/308	0.397647130794388
hsa05150	Staphylococcus aureus infection	5/308	0.397647130794388
hsa04215	Apoptosis - multiple species	4/308	0.0416810139066413
hsa05330	Allograft rejection	4/308	0.0714102309357052
hsa05332	Graft-versus-host disease	4/308	0.110577510442625
hsa05134	Legionellosis	4/308	0.20383319906
hsa05321	Inflammatory bowel disease	4/308	0.289450003355871
hsa04610	Complement and coagulation cascades	4/308	0.53106950690357
hsa04727	GABAergic synapse	4/308	0.537059993618289
hsa05032	Morphine addiction	4/308	0.556794509967911
hsa04925	Aldosterone synthesis and secretion	4/308	0.625025289764458
hsa04972	Pancreatic secretion	4/308	0.685599905210756
hsa00230	Purine metabolism	4/308	0.828084542483679
hsa04110	Cell cycle	4/308	0.936765990237965
hsa04141	Protein processing in endoplasmic reticulum	4/308	0.950272649906944
hsa04814	Motor proteins	4/308	0.972465099568449
hsa03040	Spliceosome	4/308	0.981475548710786
hsa01523	Antifolate resistance	3/308	0.139992329211903
hsa04672	Intestinal immune network for IgA production	3/308	0.3630825051
hsa05030	Cocaine addiction	3/308	0.3630825051
hsa05320	Autoimmune thyroid disease	3/308	0.412729470082765
hsa05031	Amphetamine addiction	3/308	0.609723289496843
hsa04918	Thyroid hormone synthesis	3/308	0.67214019063952
hsa03320	PPAR signaling pathway	3/308	0.680770978322578
hsa03083	Polycomb repressive complex	3/308	0.743207549010573
hsa04911	Insulin secretion	3/308	0.771680648511339
hsa00190	Oxidative phosphorylation	3/308	0.959764352415589
hsa04120	Ubiquitin mediated proteolysis	3/308	0.961421559236353
hsa04080	Neuroactive ligand-receptor interaction	3/308	0.999998748277511
hsa04136	Autophagy - other	2/308	0.448792356258077
hsa04913	Ovarian steroidogenesis	2/308	0.717882636224667
hsa04340	Hedgehog signaling pathway	2/308	0.774517565697476
hsa01212	Fatty acid metabolism	2/308	0.783398945835448
hsa04330	Notch signaling pathway	2/308	0.823226962085645
hsa00561	Glycerolipid metabolism	2/308	0.828084542483679
hsa04927	Cortisol synthesis and secretion	2/308	0.828084542483679

hsa04924	Renin secretion	2/308	0.861726215001291
hsa04971	Gastric acid secretion	2/308	0.907121830237529
hsa04721	Synaptic vesicle cycle	2/308	0.92586617155262
hsa04623	Cytosolic DNA-sensing pathway	2/308	0.93267148689231
hsa04260	Cardiac muscle contraction	2/308	0.936765990237965
hsa04970	Salivary secretion	2/308	0.955142237496484
hsa00564	Glycerophospholipid metabolism	2/308	0.959764352415589
hsa04922	Glucagon signaling pathway	2/308	0.96823632820802
hsa03013	Nucleocytoplasmic transport	2/308	0.96823632820802
hsa01240	Biosynthesis of cofactors	2/308	0.994898346758353
hsa04740	Olfactory transduction	2/308	0.999998748277511
hsa03265	Virion - Ebolavirus, Lyssavirus and Morbillivirus	1/308	0.503824298752738
hsa03450	Non-homologous end-joining	1/308	0.53106950690357
hsa00604	Glycosphingolipid biosynthesis - ganglio series	1/308	0.583627428146345
hsa00450	Selenocompound metabolism	1/308	0.630404928481569
hsa00061	Fatty acid biosynthesis	1/308	0.653514256638217
hsa00100	Steroid biosynthesis	1/308	0.685599905210756
hsa00670	One carbon pool by folate	1/308	0.685599905210756
hsa00062	Fatty acid elongation	1/308	0.798921954665739
hsa00650	Butanoate metabolism	1/308	0.798921954665739
hsa01040	Biosynthesis of unsaturated fatty acids	1/308	0.798921954665739
hsa04966	Collecting duct acid secretion	1/308	0.812275546321424
hsa00020	Citrate cycle (TCA cycle)	1/308	0.828084542483679
hsa03060	Protein export	1/308	0.828084542483679
hsa05310	Asthma	1/308	0.828084542483679
hsa04710	Circadian rhythm	1/308	0.861726215001291
hsa00250	Alanine, aspartate and glutamate metabolism	1/308	0.8906052555657
hsa01250	Biosynthesis of nucleotide sugars	1/308	0.8906052555657
hsa00380	Tryptophan metabolism	1/308	0.926786222265233
hsa04216	Ferroptosis	1/308	0.926786222265233
hsa00071	Fatty acid degradation	1/308	0.931954525332168
hsa03022	Basal transcription factors	1/308	0.93267148689231
hsa02010	ABC transporters	1/308	0.936765990237965
hsa00514	Other types of O-glycan biosynthesis	1/308	0.936765990237965
hsa00620	Pyruvate metabolism	1/308	0.936765990237965
hsa00330	Arginine and proline metabolism	1/308	0.947440322539008
hsa00520	Amino sugar and nucleotide sugar metabolism	1/308	0.947440322539008
hsa04979	Cholesterol metabolism	1/308	0.947440322539008
hsa05110	Vibrio cholerae infection	1/308	0.947440322539008
hsa04961	Endocrine and other factor-regulated calcium reabsorption	1/308	0.950272649906944
hsa00240	Pyrimidine metabolism	1/308	0.960641796636103
hsa05217	Basal cell carcinoma	1/308	0.96823632820802
hsa00970	Aminoacyl-tRNA biosynthesis	1/308	0.96823632820802

hsa00830	Retinol metabolism	1/308	0.970148648905017
hsa01230	Biosynthesis of amino acids	1/308	0.981475548710786
hsa03018	RNA degradation	1/308	0.981475548710786
hsa04612	Antigen processing and presentation	1/308	0.981475548710786
hsa00983	Drug metabolism - other enzymes	1/308	0.981475548710786
hsa04146	Peroxisome	1/308	0.981475548710786
hsa01232	Nucleotide metabolism	1/308	0.981475548710786
hsa04976	Bile secretion	1/308	0.985791234065261
hsa04974	Protein digestion and absorption	1/308	0.995302208283966
hsa01200	Carbon metabolism	1/308	0.996011623595077
hsa03082	ATP-dependent chromatin remodeling	1/308	0.996011623595077
hsa04142	Lysosome	1/308	0.998645558504815
