# Supplementary material for: A DNA methylation signature identified in the buccal mucosa reflecting active tuberculosis is changing during tuberculosis treatment
Source: Sci Rep. 2024 Nov 28;14:29552. doi: 10.1038/s41598-024-80570-4 (PMC11604703; doi:10.1038/s41598-024-80570-4)
Supplement: Supplementary file 3 — Supplementary Information 3. [file 41598_2024_80570_MOESM3_ESM.docx]

Supplementary Table 1. **TB severity score of Kenyan validation cohort.** TB score status^1,2^ at baseline (TB0) and symptoms at follow-ups after two and six months of treatment (TB2 and TB6) for Kenyan cohort. TB, tuberculosis.

| **Parameters** | **TB0 (*n*=41)** | **TB2 (*n*=25)** | **TB6 (*n*=23)** |
| --- | --- | --- | --- |
| *Self-reported* |  |  |  |
| Cough | 35 (85%) | 16 (64%) | 6 (26%) |
| Haemoptysis | 5 (12%) | 0 (0%) | 0 (0%) |
| Dyspnea | 18 (44%) | 6 (24%) | 0 (0%) |
| Chest pain | 24 (59%) | 10 (40%) | 3 (13%) |
| Night sweating | 24 (59%) | 1 (4%) | 0 (0%) |
| Anemia (anemic conjunctivae) | 9 (22%) |  |  |
| Temperature >37.8 C | 30 (73%) |  |  |
| Pulse >90/minute | 22 (54%) |  |  |
| Positive finding at lung auscultation | 18 (44%) |  |  |
| BMI <18 | 18 (44%) | 7 (28%) | 6 (26%) |
| BMI <16 | 8 (20%) | 3 (12%) | 2 (9%) |
| MUAC <220 | 22 (54%) |  |  |
| MUAC <200 | 8 (20%) |  |  |
| **Mean total TB score**^†^ | 5.9±2.2 |  |  |
| **TB severity class** | 2 |  |  |
| **Mean TB symptom score*** | 3.2 | 1.7 | 0.7 |

  1. Wejse, C. *et al.* TBscore : Signs and symptoms from tuberculosis patients in a low-resource setting have predictive value and may be used to assess clinical course. **5548**, (2009).

2. Rudolf, F. The Bandim TBscore - reliability, further development, and evaluation of potential uses. *Glob. Health Action* **7**, (2014).

Supplementary Table 2. **Additional clinical data of the Kenyan cohort.** Tuberculosis treatment regimen and treatment outcome for patients in the Kenyan validation cohort. TB, tuberculosis; 2RHZE/4RH, 2 months with rifampicin, isoniazid, pyrazinamide, ethambutol/4 months with rifampicin, isoniazid.

| **Characteristics** | **Patients (*n* = 41)** |
| --- | --- |
| *TB treatment* |  |
| 2RHZE/4RH | 41 (100%) |
| *Outcome (at 6 months)* |  |
| Cured | 15 (37%) |
| Lost to follow up or outcome unknown | 26 (63%) |
| Previous TB infection | 6 (15%) |
